# Non-redundant cardiolipin synthases shape membrane composition and support stress resilience in *Bacteroides fragilis*

**DOI:** 10.1101/2025.05.12.653583

**Published:** 2025-05-19

**Authors:** Matthew K. Schnizlein, BongJin Hong, Jennifer N.T. Nguyen, Katarina Jones, Alyssa I. Rodriguez, Aretha Fiebig, Shawn R. Campagna, Marcy J. Balunas, Thomas V. O’Halloran, Sean Crosson

**Affiliations:** 1 Department of Microbiology, Genetics & Immunology, Michigan State University, East Lansing, MI, USA; 2 Elemental Health Institute, Michigan State University, East Lansing, MI, USA; 3 Department of Medicinal Chemistry, University of Michigan, Ann Arbor, MI, USA; 4 Biological and Small Molecule Mass Spectrometry Core, University of Tennessee-Knoxville, TN, USA; 5 Department of Chemistry, University of Tennessee-Knoxville, TN, USA; 6 BioMolecular Science Gateway, Michigan State University, East Lansing, MI, USA; 7 Department of Microbiology and Immunology, University of Michigan, Ann Arbor, MI, USA; 8 Department of Chemistry, Michigan State University, East Lansing, MI, USA

## Abstract

*Bacteroides fragilis* is an anaerobic resident of the human gut known to tolerate the toxic effects of host-produced and microbially-modified bile acids. Two conserved genes, *clsA* and *clsB*, encode putative cardiolipin synthases that have been linked to bile acid tolerance, but their physiological roles remain undefined. Phylogenetic analysis indicates that *Bacteroides* spp. ClsA and ClsB diverge from the well-characterized cardiolipin synthases of Gammaproteobacteria and Firmicutes. Here, we show that these enzymes have distinct cardiolipin synthase activities and make non-redundant contributions to *B. fragilis* fitness under gut-relevant stress conditions, including osmotic stress, disruption of membrane potential, and exposure to the bile acid deoxycholate. Although deoxycholate treatment perturbed K⁺/Na⁺ homeostasis in *B. fragilis*, deletion of *clsA* or *clsB* did not significantly alter intracellular ion levels, suggesting that cardiolipin loss does not substantially impact ion balance under standard cultivation conditions. High-resolution lipidomic analyses showed that cardiolipin comprises less than 1% of *B. fragilis* membranes and that ClsA and ClsB produce distinct cardiolipin products with unique acyl chain lengths and levels of unsaturation. Deletion of either *cls* gene led to Cls-specific remodeling of *B. fragilis* envelope lipid content, which was also associated with shifts in non-lipid metabolites indicative of stress-induced metabolic changes. These results define distinct roles for ClsA and ClsB in shaping *B. fragilis* membrane composition, metabolism, and stress resilience, and highlight cardiolipin as a key determinant of fitness under bile acid stress.

## Importance

Inflammatory bowel diseases are a growing global health concern, motivating efforts to understand how inflammation-associated microbes, such as *B. fragilis*, adapt to and persist in stress conditions typical of the intestinal environment. Our targeted genetic analyses reveal that the cardiolipin synthases ClsA and ClsB make non-redundant contributions to *B. fragilis* fitness under bile acid exposure and other gut-relevant stress conditions. Complementary omics-based approaches show that these enzymes produce chemically distinct cardiolipin species and that their loss leads to specific remodeling of membrane lipid composition and cellular metabolite profiles. Together, our results indicate that cardiolipin synthases contribute to membrane and metabolic adaptation in *B. fragilis*, supporting stress resilience in the presence of bile acids and other gut-relevant stressors.

## INTRODUCTION

To successfully occupy gut niches and maintain homeostasis, bacteria must preserve membrane integrity to resist phage attack, host immune pressures, and membrane-disrupting molecules such as bile acids (1–7). The functional properties of membranes are shaped by the diverse lipids classes of which they are made, each characterized by an array of structural species (8–10). For example, bacteria can alter their membrane fluidity and/or permeability by modulating the length of phospholipid acyl tails in response to extracellular conditions (8, 11). Bacteria also adjust the abundance of different phospholipid types or modify lipid headgroups through reactions such as aminoacylation (12–16).

In addition to protecting against external threats, membranes also help support cellular metabolism by maintaining a potential energy source in the form of ion gradients, such as hydrogen (protons, H^+^), sodium (Na^+^), and potassium (K^+^) (17). One of the best-known functions of proton gradients is to power rotary ATPase complexes (i.e., F_o_F_1_) associated with aerobic and anaerobic respiration (18–22). Prokaryotes as well as eukaryotes maintain Na^+^ and K^+^ at intracellular concentrations that are lower and higher, respectively, relative to their environment, which drives the transport of metals or other molecules across the membrane (23, 24).

Cardiolipins, which make up 1–5% of the bacterial membrane, play an integral role in maintaining ion gradients (25–30). This class of membrane lipid comprises two covalently bonded phospholipids, such as phosphatidylglycerol (PG) or in some cases phosphatidylethanolamine (PE) (31). While cardiolipins increase proton permeability across model liposome membranes (28, 32), they also contribute to membrane stabilization by enhancing hydrogen bonding, reducing water permeability and sustaining membrane potential during stress-induced membrane depolarization (32, 33). In addition, several protein complexes require cardiolipin for proper function, including some two-component systems, protein translocation machinery, and respiratory complexes (34–39).

*Escherichia coli* has three well-studied genes responsible for cardiolipin biosynthesis: *clsA*, *clsB* and *clsC* (31, 40). Each gene makes distinct contributions to *E. coli* physiology depending on growth phase, with *clsA* being the primary *cls* used during logarithmic (log)-phase growth and all three used during stationary phase (26, 31). While ClsA and ClsB act on PG as the sole substrate, ClsC synthesizes cardiolipin from PE as well as PG (31, 41–43). The Cls enzymes encoded by these genes are thought to synthesize three structural families of cardiolipin, which have four (cardiolipin), three (monolysocardiolipin) and two acyl tails (dilysocardiolipin), respectively (for chemical structures, see Fig. 6A) (44). However, mechanistic insight into bacterial cardiolipin biosynthesis and function largely comes from studies in *E. coli* and other Enterobacteriaceae. Expanding this research across diverse bacterial lineages is essential for uncovering the broader physiological roles of this important lipid class.

The gut microbe *Bacteroides fragilis* is a gram-negative facultative anaerobe that must maintain membrane integrity for gut fitness (45–47). While *B. fragilis* strains, particularly those that carry the fragilysin toxin, are linked to colorectal cancer and other diseases (48), it constitutes about 1–5% of the microbiota in otherwise healthy humans (49, 50). We recently identified two cardiolipin synthases as critical for the survival of the non-toxigenic *B. fragilis* strain P207 in the presence of physiologically relevant levels of deoxycholate, a secondary bile acid (51). *B. fragilis* P207 was isolated from the ileoanal pouch of a patient with ulcerative colitis (UC) and is representative of *B. fragilis* populations that can dominate prior to the onset of pouchitis (52), a condition characterized by UC-like inflammation of the ileoanal pouch following colectomy (53). In this study, we build on our previous findings by investigating the roles of the two *B. fragilis* cardiolipin synthases across a broader range of membrane stress conditions. Since cardiolipin synthases are largely uncharacterized in the Bacteroidota, our work provides foundational knowledge about this important class of enzymes in a dominant group of gut microbes (54). Using a combination of approaches, we demonstrate that the two *cls* genes in *B. fragilis* P207, *clsA* and *clsB*, are evolutionarily and functionally distinct from *cls* homologs in *E. coli* and other well-studied bacterial phyla. These genes encode non-redundant enzymes that contribute structurally unique cardiolipin species to the membrane, conferring distinct physiological advantages under stress. We further show that *clsA* and *clsB* differ in their expression profiles and that mutants lacking each gene produce distinct cardiolipin species as well as distinct metabolomic profiles, supporting complementary roles in maintaining membrane homeostasis and adapting to diverse environmental conditions.

## RESULTS

### B. fragilis *P207 ClsA and ClsB are cardiolipin synthases*

**Figure 1.**
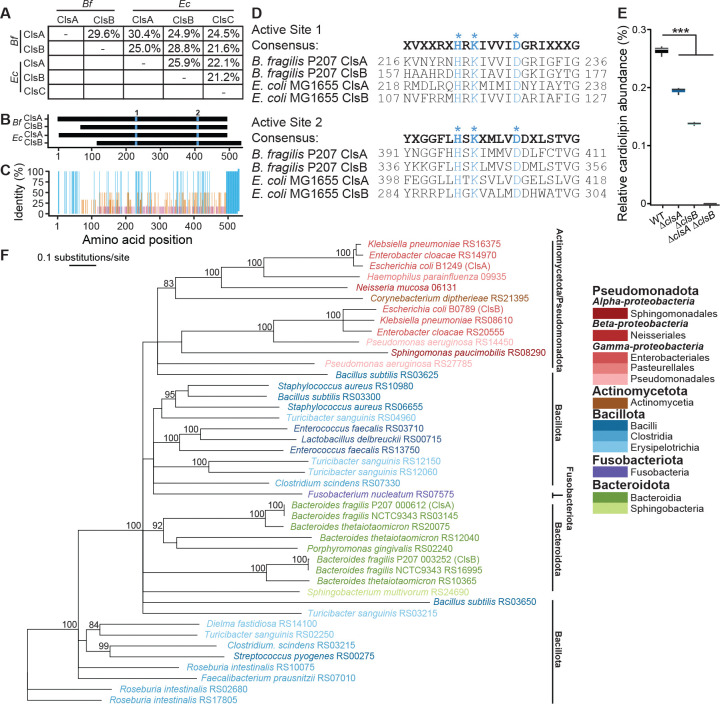
Cardiolipin synthases diverge across the dominant gut bacterial phyla. **A.** Percentage identity from pairwise amino acid alignment of cardiolipin synthases in *B. fragilis* P207 (*Bf*) and *E. coli* K12 MG1655 (*Ec*). **B.** Alignment of ClsA and ClsB from *B. fragilis* P207 and *E. coli* MG1655, with the two HKD domains marked in blue. **C.** Percentage identity from amino acid alignment in panel B colored based on the thresholds <30% (pink), <100% (orange) and 100% (blue). **D.** Consensus and individual sequences of the regions flanking the two HKD active sites of *B. fragilis* and *E. coli* Cls proteins. HKD residues are colored in blue; the N- and C-terminal positions are shown. **E.** Relative abundance of cardiolipin in *B. fragilis* P207 wild-type (WT), Δ*clsA*, Δ*clsB* and Δ*clsA ΔclsB* strains measured by mass spectrometry. The horizontal line indicates the median, and the whiskers 1.5× the interquartile range outside the 25^th^ and 75^th^ percentiles of n = 3 samples. (linear regression; unadjusted p-values are shown; *** indicates *p* < 0.001). **F.** Neighbor-joining tree of a Cls alignment from representative taxa of major bacterial phyla in the gut, using only one genome per species. For each sequence in the tree, taxa names are followed by genome IDs at the tip of each branch; genome accession numbers and other metadata are provided in Table S2. Bootstrap values are shown only if >80%. Bacterial taxa are colored according to the phylum to which they belong (red, Pseudomonadota – 3 classes with 1, 1, and 3 families; brown, Actinomycetota – 1 class; blue, Bacillota – 3 classes; violet, Fusobacteriota – 1 class; green, Bacteroidota – 2 classes, respectively). Key indicates the rate of amino acid substitutions per site.

The two *B. fragilis* P207 genes, ptos_000612 (*clsA*) and ptos_003252 (*clsB*), encode proteins with predicted cardiolipin synthase domains (TIGR04265, PFAM13091, cd09112), though *clsA* and *clsB* have low nucleotide (52.4%) and amino acid sequence (29.6%) identity to each other (Fig. 1A). The low primary structure identity (≤30%) of ClsA and ClsB to *E. coli* Cls proteins limited our ability to predict their specific enzymatic activities based on sequence alone (Fig. 1A). Furthermore, *E. coli* ClsC did not align well with ClsA or ClsB from either *B. fragilis* or *E. coli* (Fig. 1A, B). We therefore excluded ClsC from further comparisons.

ClsA and ClsB from *B. fragilis* and *E. coli* exhibit high amino acid conservation in two regions around their predicted active sites but share limited identity across the remainder of the protein (Fig. 1B, C). The Cls proteins from both species harbor two phospholipase-D active site motifs H-X-K-X(4)-D (26, 42), indicative of phospholipase-D activity (Fig. 1D). Indeed, deleting either *clsA* (Δ*clsA*) or *clsB* (Δ*clsB*) in *B. fragilis* resulted in a partial loss of cellular cardiolipin accumulation relative to the wild-type (WT) strain; deleting both genes (Δ*clsA* Δ*clsB*) completely abolished cardiolipin accumulation (Fig. 1E). We conclude that *clsA* and *clsB* in *B. fragilis* P207 encode *bona fide* cardiolipin synthases. A more comprehensive analysis of how deletion of *clsA* and *clsB* affects the membrane lipid composition of the *B. fragilis* envelope is presented in sections below.

### *The cardiolipin synthases* of Bacteroides *sp. occupy a distinct phylogenetic cluster among dominant gut bacterial phyla*

To evaluate the relatedness between *Bacteroides* Cls proteins and those encoded by other gut microbes, we performed pairwise alignments of Cls sequences from dominant bacterial phyla present in the human gut. These sequences clustered according to their evolutionary heritage, except for the Bacillota, which had two clusters (Fig. 1F). Based on this result, we conclude that each cardiolipin synthase originated from a proto-lineage, rather than having been horizontally transferred.

We next evaluated Cls protein sequences within the *Bacteroides* genus. We aligned 301 *Bacteroides* protein sequences containing the TIGR04265 domain (i.e. cardiolipin synthases). Most genomes encoded two cardiolipin synthases that partitioned into distinct clusters (Fig. S1). Notably, the *B. thetaiotaomicron* genome encodes a third Cls that is distinct from the other two *Bacteroides* Cls clusters (Fig S1) indicating an evolutionarily distant introduction. We designate cluster 1 homologs as ClsA based on our observation that both *B. fragilis* and *E. coli* ClsA proteins are predicted to have two N-terminal transmembrane helices, suggesting a similar mode of post-translational processing (55, 56). We named the cluster 2 homologues ClsB, as they represent proteins from the second and only other annotated *cls* gene in most *Bacteroides*. We left the third *cls* gene, which we identified only in *B. thetaiotaomicron*, unnamed and did not further characterize it.

Within the *Bacteroides* genus, the chromosomal gene neighborhoods around *clsA* (Fig. S2A) and *clsB* (Fig. S2B) are highly conserved; this conservation is lost outside the *Bacteroides*. Thus, the genomic context of *clsA* and *clsB* provides some indication of the identity of each *cls* gene, specifically within the *Bacteroides* lineage. This evidence also supports the hypothesis that taxa inherited each *cls* gene in two distinct regions of the chromosome. Considering this phylogeny, our analysis of the *B. fragilis cls* genes presented below offer insight into the function of the two broadly distributed cardiolipin synthases across the genus *Bacteroides*.

### clsA *and* clsB *deletion mutants have growth defects under membrane stress*

**Figure 2.**
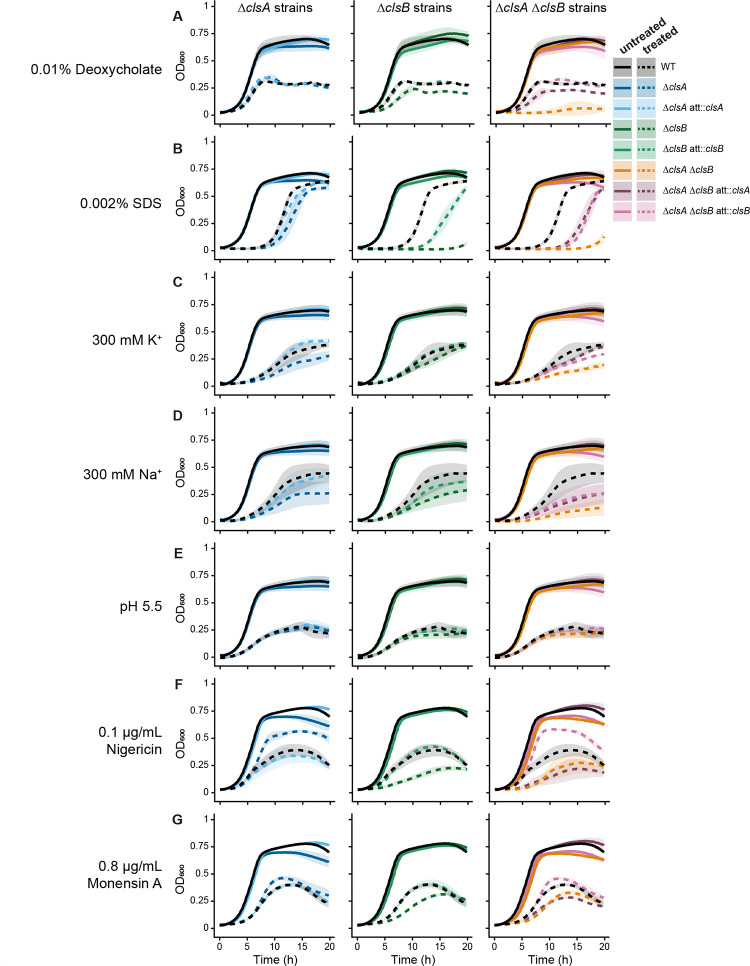
Loss of cardiolipin synthase activity results in growth defects under stress. Optical density (OD_600_) measurements of *B. fragilis* P207 wild-type (WT) and *cls* single or double mutant strains exposed to the detergents (**A.**) 0.01% deoxycholate or (**B.**) 0.002% SDS; the salts (**C.**) 300 mM K^+^ or (**D.**) 300 mM Na^+^; (**E.**) medium adjusted to pH 5.5; and the ionophores (**F.**) 0.1 μg/mL nigericin or (**G.**) 0.8 μg/mL monensin A. Untreated (solid lines) and treated cultures (dashed lines) are plotted by strain, with blue, green and orange/purple indicating *clsA*-, *clsB*- and *clsA clsB*-related strains, respectively. Black indicates WT. The shaded areas around each line indicate 2× the standard error of the mean of n = 3 biological replicates.

To explore the functions of the two cardiolipin synthases in *B. fragilis* P207, we generated mutant strains harboring in-frame deletions of *clsA* (Δ*clsA*) and *clsB* (Δ*clsB*), and a strain lacking both *cls* genes (Δ*clsA ΔclsB*). We also generated corresponding genetic complementation strains bearing *clsA* or *clsB* integrated at a neutral heterologous site in the chromosome. We assessed the growth characteristics of these *B. fragilis* strains under stress conditions that perturb various aspects of membrane biology (Fig. 2). The treatments in this study included: 1) the detergents deoxycholate and sodium dodecyl sulfate (SDS); 2) low pH; 3) the salts NaCl and KCl; and 4) the ionophores monensin A (for Na^+^) and nigericin (for K^+^ and H^+^) to disrupt transmembrane ion gradients. Treatment concentrations were chosen based on their effect on WT strain growth.

In the absence of treatment, all strains grew equally well (Figs. 2A, S3A, B). Treatment with the detergents deoxycholate (at 0.01% [w/v]; Fig. 2A, S3) or SDS (at 0.002% [w/v]) (Fig. 2B) limited the growth of the *cls* mutant strains to a greater extent than WT. Deoxycholate affected maximum OD_600_ in the Δ*clsA ΔclsB* att::*clsA* strain, and both max OD_600_ and growth rate the Δ*clsA ΔclsB* strain. SDS treatment mainly affected lag time, decreasing the max OD_600_ values in the first 20 h of growth. Doubling time defects appeared as little or no growth, rather than slower growth (Fig. 2B). Under both detergent conditions, the deletion of *clsB* had a larger effect on fitness than deletion of *clsA* (Figs. 2A, B, S3) and deletion of both genes (Δ*clsA* Δ*clsB*) had an additive effect. 300 mM K^+^ or Na^+^ (Fig. 2C, D) also lowered the growth rate and maximum OD_600_ of the mutant strains relative to WT (Fig. S3). Both Δ*clsA* and Δ*clsB* strains exhibited similar phenotypes, with the Δ*clsA ΔclsB* strain showing more pronounced effects. Lowering the medium pH from 6.9 to pH 5.5 (i.e., higher [H^+^]) had a detrimental effect on all *B. fragilis* strains, with no clear *cls*-dependent growth defects (Fig. 2E).

The ionophores nigericin and monensin A dissipate ion gradients by mediating the exchange of ions across the inner membrane (57, 58). Specifically, nigericin is a potassium-selective ionophore and a H^+^-K^+^ antiporter, erasing both gradients by acidifying the cytoplasm and moving K^+^ out of the cell (57). Monensin A is a sodium-selective ionophore with a 16-fold preference for Na^+^ over K^+^, but can also affect H^+^ gradients (57–59). Under either nigericin or monensin A treatment, *clsB* deletion decreased strain fitness relative to the WT (Fig. 2F, G). However, strains harboring *clsB* but lacking *clsA* (Δ*clsA* and Δ*clsA ΔclsB* att::*clsB*) exhibited a higher maximum OD_600_ relative to the WT (Fig. S3).

We conclude that deleting *clsB* results in greater fitness defects than loss of *clsA* under membrane perturbations. Furthermore, a functional ClsA limits growth under nigericin and monensin A treatment, as we observed increased culture density in the Δ*clsA* strains. These growth phenotypes provide evidence that ClsA and ClsB have distinct roles in *B. fragilis* membrane physiology.

### Loss of cardiolipin changes cell shape

**Figure 3.**
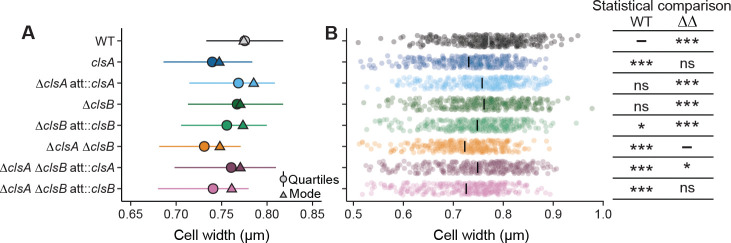
Loss of cardiolipin synthase activity is associated with defects in cell width. **A.** Summary of the cell width distributions. Horizontal line lengths indicate the 25% and 75% quartiles of the distribution; circles indicate the median; triangles indicates the mode (i.e., the peak of the distribution). **B.** Cell width data for all cells shown in A. plotted by strain (*n* = 323 cells/strain). The gray or black bars indicate the mean. The table at right shows the results of a mixed-effect linear model comparing strain width to the widths of either WT cells or Δ*clsA* Δ*clsB* double mutant (ΔΔ) cells, as indicated at the top of the column, with a random intercept of biological replicate (i.e., cell width ~ strain + 1|biological replicate). Unadjusted p-values are shown; *, *p* < 0.05, *** *p* < 0.001.

Given that in many bacteria, cardiolipin influences cell division, morphology, and membrane fluidity (60–67), we asked whether loss of Cls function in *B. fragilis* would affect cell shape. Deletion of either *cls* gene did not markedly affect overall rod-like cell shape or cellular length distribution (Fig. S4A, B). However, cells lacking *clsA* (i.e., Δ*clsA* and Δ*clsA ΔclsB* strains; Figs. 3A, B and S4C) were narrower. While cells lacking *clsB* were not significantly narrower than WT, this cell population did have a broader cell width distribution compared to WT, suggesting a role for ClsB in cell size regulation (F-test, WT to Δ*clsB p* = 0.019, WT to Δ*clsA ΔclsB att:clsA p* < 0.001, WT variances compared to Δ*clsA* and Δ*clsA ΔclsB* were not significantly different; Figs. 3A and S4C). Both phenotypes could be genetically complemented. These results demonstrate that ClsA and ClsB influence cell width in *B. fragilis* (Fig. 3A, B).

### B. fragilis *cardiolipin synthase genes have distinct expression profiles*

**Figure 4.**
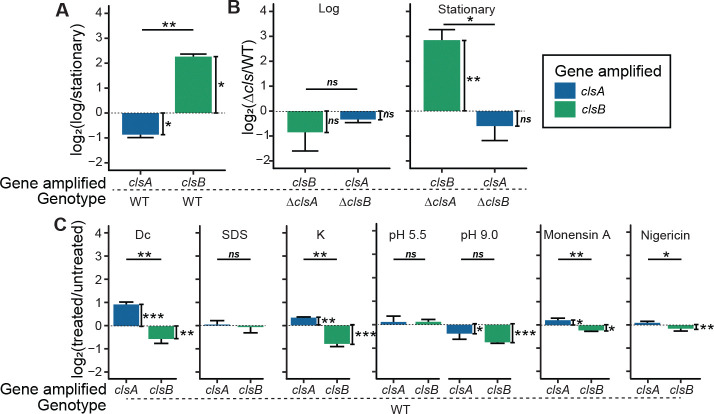
Cardiolipin synthase genes have distinct expression patterns. **A.** RT-qPCR analysis of relative levels of *clsA* and *clsB* transcripts, shown as the log_2_ ratios between log- and stationary-phase growth. **B.** Expression levels of *clsA* or *clsB* when the other gene is knocked out in log- or stationary-phase growth. **C.** *clsA* and *clsB* expression levels in WT *B. fragilis* P207 after 20 min exposure to of 0.01% deoxycholate, 0.002% SDS, 300 mM K, pH 5.5, pH 9.0, 0.8 μg/mL monensin A, or 0.1 μg/mL nigericin. For all panels, values are means ± standard deviation from three biological replicates, each biological replicate value being the mean of three technical replicates. The vertical brackets for statistical analysis assess the significance of the difference between conditions in A and C, between mutant and WT in B. The horizontal brackets for statistical analysis assess the significance of the difference between ΔΔCt values for *clsA* and *clsB* (Students *t*-test). Genes amplified are colored in blue (*clsA*) or green (*clsB*). Error bars indicate standard deviation. Unadjusted p-values are shown; *, p < 0.05; **, p < 0.01; ***, p < 0.001.

Considering the unique phenotypes of the Δ*clsA* and Δ*clsB* mutant strains, we hypothesized that these genes have distinct expression patterns. To test this idea, we quantified *clsA* and *clsB* transcripts levels across growth phases and stress conditions. *clsB* transcript levels were four-fold higher during logarithmic (log) phase growth than in stationary phase, while *clsA* expression was two-fold higher in stationary phase (Fig. 4A). During logarithmic growth, deletion of either *cls* gene did not affect the transcript levels of the other (Fig. 4B). However, in stationary phase, the deletion of *clsA* led to an 8-fold increase in *clsB* transcripts. No change in *clsA* transcripts were observed upon *clsB* deletion (Fig. 4B). We conclude that the two cardiolipin synthase genes of *B. fragilis* have distinct, growth-phase dependent expression profiles and that *clsB* transcript upregulation in the Δ*clsA* strain may compensate for loss of ClsA activity.

To test if membrane stress influences the expression of the *cls* genes, we subjected cells to acute stress for 20 minutes and harvested RNA. While SDS treatment did not affect *cls* transcript levels, we observed a two-fold increase in *clsA* expression and a small decrease in *clsB* transcripts after 0.01% deoxycholate and 300 mM K^+^ treatments (Fig. 4C) (51). Acidic pH did not alter *cls* transcript levels, but basic pH did decrease transcript levels of both genes (Fig. 4C). Finally, we detected differences under ionophore treatment, although the biological significance of these smaller changes is unclear (Fig. 4C).

### Loss of cardiolipin synthase genes does not have a major impact on homeostasis of major cellular elements

As cardiolipins play an important role in maintaining membrane barrier integrity (25–27, 68, 69), we used inductively coupled plasma mass spectrometry (ICP-MS) to quantitatively define amounts of certain elements in *B. fragilis* cells and measure the effect of ClsA and/or ClsB or stress treatment on those elements. While cell washing is routinely used to prepare samples for metal analysis, it can disrupt intracellular levels of elements (Fig. S5A). Therefore, we used a wash-free ICP-MS assay to minimize perturbation during sample preparation, adapted here for bacterial cultures (70). Using this approach, we found that *B. fragilis* P207 cells grown in supplemented brain heart infusion (BHIS) medium contained 550 mM phosphorus (P), 430 mM potassium (K), 98 mM sulfur and 63 mM sodium (Na) (Fig. S5C, medium elemental concentrations shown in S5B).

**Figure 5.**
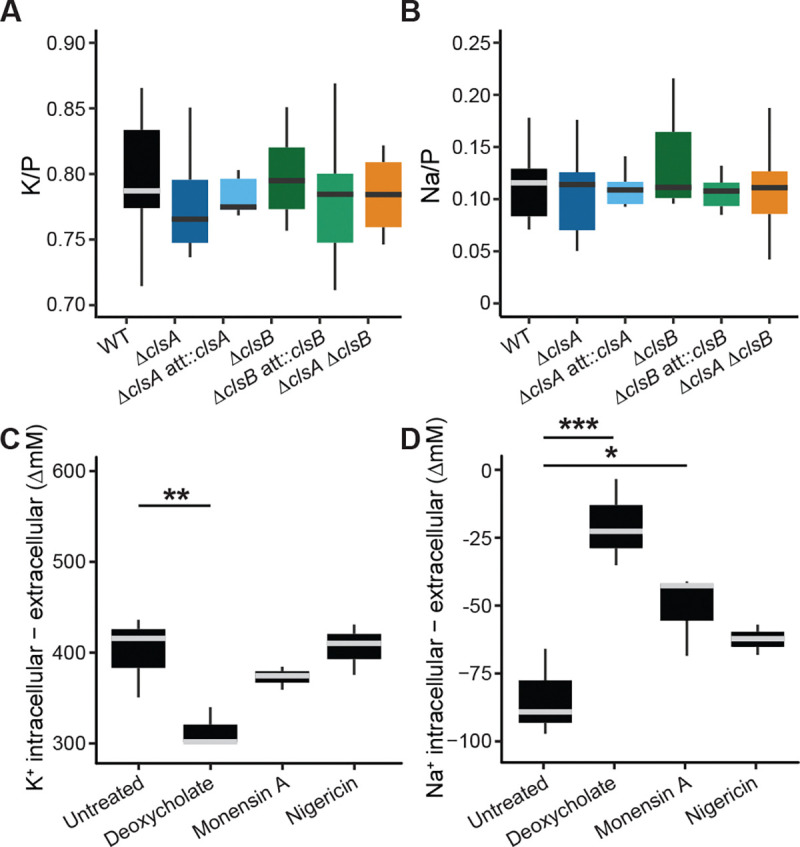
Stress but not loss of Cls function disrupts membrane ion gradients. **A., B.** Abundance of (**A.**) potassium (K) and (**B.**) sodium (Na) normalized to intracellular phosphorus (P) in *B. fragilis* P207 WT, Δ*clsA*, Δ*clsB* and complemented strains as well as the Δ*clsA ΔclsB* strain, as detected by inductively coupled plasma mass spectrometry (ICP-MS). **C., D.** Differential concentrations of (**C.**) K^+^ and (**D.**) Na^+^ in WT strain cells after a 20 min exposure to 0.01% deoxycholate, 0.8 μg/mL monensin A or 0.1 μg/mL nigericin (linear regression). The intracellular concentrations (i.e., normalized to cell volume and number of colony-forming units) with the extracellular concentrations subtracted are shown. For all boxplots, gray or black lines indicate the median; the top and bottom edges of the box indicate the 25^th^ and 75^th^ percentiles of the data; the error bars indicate standard deviation (10 biological replicates for untreated conditions and three for the treatment conditions). Unadjusted p-values are shown; *, p < 0.05; **, p < 0.01; ***, p < 0.001.

To compare strains, we normalized intracellular element levels to phosphorus content, an accepted practice in this type of analysis (71). This revealed no significant differences across the Δ*clsA*, Δ*clsB*, or double-deletion strains in the relative levels of elements detected (Fig. 5A, B, and S5C). Notably, given the lack of strain-dependent defects in CFU viability, we observed shifts in relative phosphorus content per CFU across *cls* mutants that was consistent with differences in the OD_600_/CFU ratios (Fig. S5E–G). Furthermore, sulfur levels scaled proportionally with phosphorus across samples (Fig. S5D and S5H). While P and S abundance can vary across physiological states of the cell, they are tightly regulated (72, 73). Together, the elemental composition and OD_600_ data suggest that *cls* deletion can alter the ability of *cls* deletion strains to consistently form colonies.

Like most organisms, *B. fragilis* maintains a higher concentration of intracellular K^+^ (+400 ΔmM) and lower concentration of intracellular Na^+^ (−67 ΔmM) than the surrounding environment (Fig. 5C, D). Exposure to deoxycholate, a microbially-modified bile acid, led to decreased intracellular K^+^ and increased Na^+^ contents, providing evidence that this treatment indeed disrupts transmembrane ion gradients in *B. fragilis* (Figs. 5C, D & S5I–J). The observed increase in intracellular P per CFU (Fig. S5K) suggests that some of these changes could result from the presence of non-viable cells (Fig. S5L), as these cells would retain the S and P mass associated with large molecules like proteins and nucleotides, while smaller, more permeable elements would be lost across damaged membranes (74, 75). Treatment with the sodium selective ionophore, monensin A, did not affect K^+^ levels but caused a 32 mM increase in intracellular Na^+^ (Fig. 5C, D). By contrast, treatment with the potassium selective ionophore, nigericin, caused no detectable effect on either K^+^ or Na^+^ content, suggesting that nigericin may require more than 20 min to act on *B. fragilis*, or that the cells quickly adapt to the imposed ionic disturbance (Fig. 5C, D).

### *Membrane lipid composition of WT* B. fragilis *P207*

**Figure 6.**
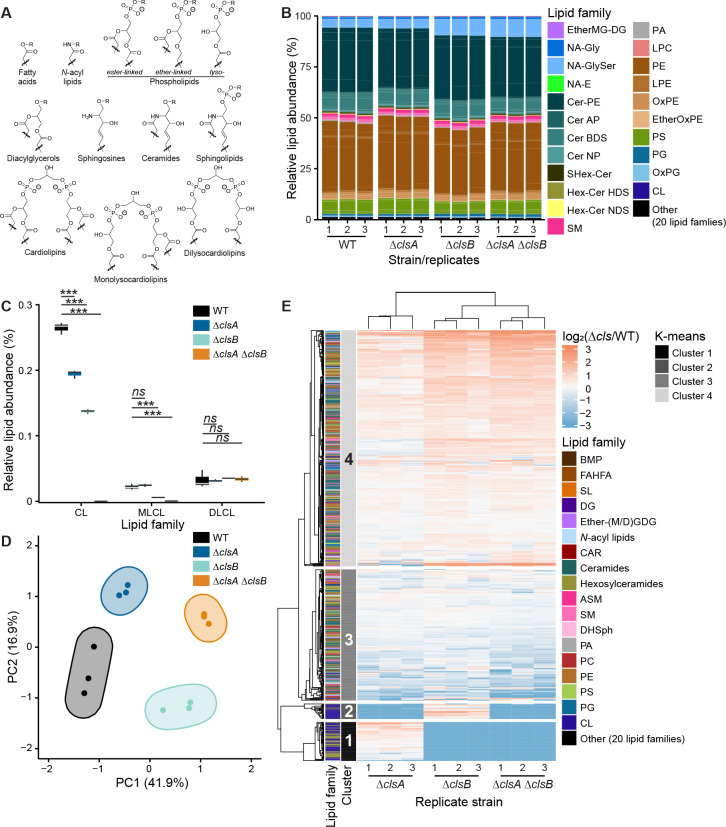
Paralogous cardiolipin synthases produce unique lipid products. **A.** Structures of lipid families detected by UHPLC-MS/HRMS. For fatty acids, the R-group is H; for saccharolipids, a sugar (i.e., lipid A); and for acyl carnitines, a carnitine. The R-group is H for phosphatidic acid and ethanolamine for phosphatidylethanolamine. Phospholipids are shown as members of three categories: ester-linked (i.e., classical phospholipids), ether-linked and lysophospholipids. Diacylglycerols and monogalactosyl / monoglucosyl-diacyglycerols have H or a hexose ring as an R-group, respectively. Ceramides, which can be conjugated to a hexose (i.e., hexosylceramide), are synthesized by *N*-acylating a sphingosine. As phosphorylated ceramides, sphingolipids have similar R-groups as phospholipids. Length and unsaturation of the acyl tails are not shown. **B.** Relative abundance of lipid species across WT, Δ*clsA, ΔclsB and ΔclsA ΔclsB* strains (three biological replicates). Lipid families are abbreviated as follows: PC indicates phosphatidylcholine; PA, phosphatidic acid; PE, phosphatidylethanolamine; PG, phosphatidylglycerol; PS, phosphatidylserine; CL, cardiolipin. L-, ML- and DL- indicate lyso-, monolyso- and dilyso-, respectively. **C.** Relative abundance of cardiolipin, monolyso-cardiolipin and dilysocardiolipin (linear regression). Unadjusted p-values are shown; ***, p < 0.001. **D.** Principal component analysis plot of lipid species in each strain, with the minimum volume–enclosing ellipses estimated by the Khachiyan algorithm. **E.** Heatmap representation of the log_2_(fold change) of lipid species in each knockout strain compared to WT. Columns are clustered by similarity and rows by K-means. The left side of the heatmap shows lipid families and K-means clusters.

Through tandem mass spectrometric-based lipidomics (MS/MS), we characterized lipid species present in *B. fragilis* P207 membranes using both negative and positive electrospray ionization modes. To get an overall picture of WT cell membranes, we calculated the relative peak area abundance for lipids detected in negative ionization mode. This analysis revealed that WT cell membranes were predominantly composed of phospholipids (47% of total peak areas) and ceramides (42%). The remaining abundance consisted of saccharolipids, diacylglycerols and *N*-acyl lipids (Figs. 6A & S6A). The major phospholipids were species of PE (37% of total peak areas), PS (7.2%) and PG (1.5%) (Fig. 6A, B, Table S3). Cardiolipin species accounted for approximately 0.33% of membrane lipids detected (Fig. 6B, C). Dominant ceramides were ceramide phosphoethanolamine (31.7% of total peak area) and ceramide beta-hydroxy fatty acid-dihydrosphingosine (8.6%). These results are consistent with previous studies on *Bacteroides* membrane lipid contents (76–78).

Since cardiolipin synthases use phospholipids as substrates, we investigated the types of PA, PE, PS and PG species. For PA, PS and PE, the dominant lipid types were fully saturated (30:0), comprising 85.8%, 60.7% and 57.1% of their respective classes (Fig. S6B–C). The most prevalent PG species was 32:0 (54.5%, Fig. S6B). *B. fragilis* membranes also contained lysophospholipids at lower levels than their two-acyl tailed versions (Fig. 6A), the most abundant species of which were fully saturated at C_15_ chains for lysophosphatidylethanolamine (LPE; 76.9%) and lysophosphatidylserine (LPS; 54.9%), and unsaturated at 18:3 for lysophosphatidic acid (LPA; 70.5%) (Fig. S6D). Ether-linked lipids and cardiolipin lipid pools showed much greater diversity in their constituent acyl chains (Fig. S6E–J).

### clsA *and* clsB *mutants have distinct cardiolipins profiles*

We compared the membrane lipid profiles of the *B. fragilis* P207 WT strain and the derived Δ*clsA*, Δ*clsB*, and Δ*clsA* Δ*clsB* mutant strains (Fig. 1E). While the overall abundance of the dominant lipid classes did not vary significantly among the WT and *cls* mutant strains (Fig. 6B), abundances of cardiolipin and monolysocardiolipin were lower in the *cls* deletion mutants (CL and MLCL; Fig. 6C). Total cardiolipin levels were lower in the Δ*clsA*, Δ*clsB*, and Δ*clsA* Δ*clsB* strains compared to WT, with the double knockout strain having no measurable cardiolipin (Fig. 6C). We detect lower MLCL levels only in strains lacking *clsB*, indicating that ClsB is primarily responsible for their biosynthesis (Fig. 6C). Notably, the deletion of both *clsA* and *clsB* led to no change in the detected dilysocardiolipin (DLCL), indicating that in contrast to *E. coli* where Cls proteins synthesize these lipids, a different enzymatic pathway is responsible for biosynthesis of DLCLs in *B. fragilis* (Figs. 6C and S6K) (31, 41).

**Figure 7.**
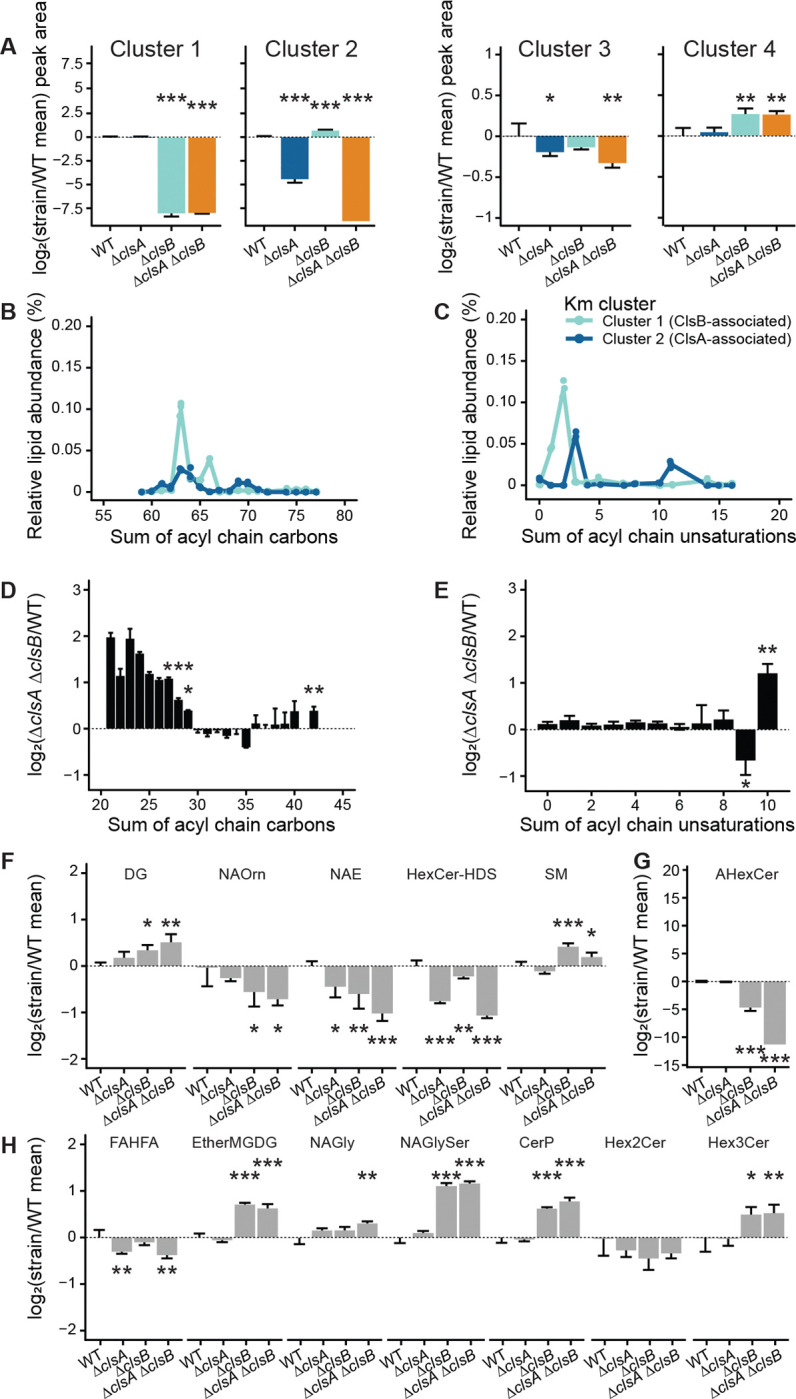
Cardiolipin synthases shape the length and unsaturation level of membrane lipid acyl chains. **A.** log_2_(fold change) of the total peak areas for all lipids in k-means clusters compared to the mean of WT peak areas of that particular cluster (linear regression of peak areas compared to WT). **B., C.** Relative distribution of sum of acyl chain (**B.**) carbons and (**C.**) unsaturations in cardiolipin and monolysocardiolipin species of Km clusters 1 (ClsB-associated) and 3 (ClsA-associated) in WT cells. **D., E.** log_2_(fold change) of total two acyl-tailed phospholipids in Δ*clsA ΔclsB* strain cells compared to WT shown by (**D.**) acyl chain length and (**E.**) unsaturations. Student’s *t*-test comparing values at designated x-axis values); nd, not detected (i.e., 0) in the Δ*clsA ΔclsB* strain. **F., G., H.** log_2_(fold change) of lipids detected in negative (**F.**) and positive (**G., H.**) ionization mode UHPLC-MS/HRMS comparing each strain to the mean WT peak area. AHexCer was detected in only 2 and 1 replicates of Δ*clsB* and Δ*clsA ΔclsB*, respectively. Lipid families are abbreviated as follows: DG, diacylglycerol; MG, monogalactosyl/monoglucosyl conjugation; NA, *N*-acyl, with substituent conjugations indicated for glycyl- (Gly), glycylseryl- (GlySer, ornithine- (Orn), and ethanolamine- (E); CER, ceramide, with A indicating acyl-, P indicating phospho- and Hex, Hex2 and Hex3 indicating hexosyl-, dihexosyl-, and trihexosyl-; HDS, hydroxy fatty acid-dihydrosphingosine; SM, sphingomyelin; Ether-, ether-linked (linear regression compared to WT). Across all panels, error bars indicate standard deviation. Unadjusted p-values are shown; *, *p* < 0.05; **, *p* < 0.01; ***, *p* < 0.001.

Principal component analysis (PCA) of membrane lipid peak areas revealed that each mutant strain clustered separately, indicating that ClsA and ClsB independently shape membrane lipid composition, while the double knockout reflected the combined loss of both functions (Fig. 6D). K-means clustering of lipid changes in *cls* deletion mutants relative to WT identified four distinct lipid subpopulations (Fig. 6E). Clusters 1 and 2 were composed almost entirely of cardiolipin, monolysocardiolipin and hexosylceramides (i.e., cluster 1 = AHexCer, cluster 2 = AHexCer, Hex2Cer, HexCer_HDS), and were absent in the Δ*clsA* Δ*clsB* double deletion strain. On average, the peak area of lipids in cluster 3 was significantly lower in strains lacking ClsA, while cluster 4 lipids were significantly higher in strains lacking ClsB (Fig. 7A). Lipids from cluster 1 were largely missing is strains without *clsB*, and lipids from cluster 2 were largely missing in strains without *clsA*. We therefore refer to these as *clsB*- and *clsA*-associated lipid clusters, respectively. From these results, we conclude that ClsA and ClsB catalyze the production of distinct lipid species in *B. fragilis*, consistent with the unique phenotypes observed in their respective deletion mutants (Fig. 2).

### ClsA and ClsB differentially influence cardiolipin saturation and membrane composition

To better understand the roles of ClsA and ClsB in cardiolipin biosynthesis, we analyzed the acyl chain length and degree of unsaturation in CL and MLCL species from lipid clusters 1 and 2. The cardiolipins associated with ClsA and with ClsB exhibited a different distribution of chain lengths, including a subset of longer species that were only detected when ClsB was present (Figs. 7B and S7A). The major differences between ClsA- and ClsB-associated lipids were in acyl chain unsaturation: ClsA-associated cardiolipins were enriched in species with 3 and 11 double bonds, whereas ClsB-associated cardiolipins were enriched with 1, 2, and 14 double bonds (Fig. 7C & S7B). These unique lipid profiles provide evidence that ClsA and ClsB have distinct substrate preferences.

To determine whether *cls* deletions alter the membrane lipidome globally, we performed PCA on the lipid profiles of each sample excluding cardiolipins. This analysis showed that each strain still clustered separately with an additive phenotype, suggesting broad ranging, *cls-*specific changes (Fig. S7C). Upon loss of cardiolipin biosynthesis in *B. fragilis*, we observe altered membrane lipid composition, including shortening the acyl chain length of phosphatidylethanolamine (PE), phosphatidylglycerol (PG) and phosphatidylserine (PS) species (Figs. 7D and S7D). This shift was coupled with increases in lipid acyl chains with a high number of total unsaturations (Figs. 7E and S7E). The combined effect of shorter acyl chains and increased unsaturation is predicted to alter membrane fluidity and permeability, potentially compensating for cardiolipin loss. Additional remodeling occurred in non-phospholipid lipid classes, with lipid families such as fatty acids (FAHFA) and hexosylceramides (HexCer-HDS) more affected by *clsA* deletion, while *N*-acyl lipids (NAOrn, NAE, NAGlySer), phosphoceramides (CerP), other hexosylceramides (AHexCer, Hex3Cer), and ether-linked glycolipids (EtherMGDG) were more affected by *clsB* deletion (Fig. 7F–H & S7F–G). These patterns were confirmed by elastic net regression, which distinguished the double mutant from WT based reductions in Cluster 1 (CL 75:3, AHexCer [O-22:6]51:9:2O) and Cluster 3 (PE O-38:0:2O) lipid species, as well as increases in two Cluster 4 lipids (NAGlySer 29:0:1O, PC O-24:4:1O). Together, these results provide evidence that ClsA and ClsB have distinct biosynthetic activities that contribute to cardiolipin diversity, and that the loss of either enzyme perturbs the membrane lipidome in unique ways. These broader shifts in membrane composition upon *cls* gene deletion likely underlie the distinct phenotypes observed in each mutant strain.

### ClsA and ClsB loss lead to distinct changes in non-lipid metabolites

**Figure 8.**
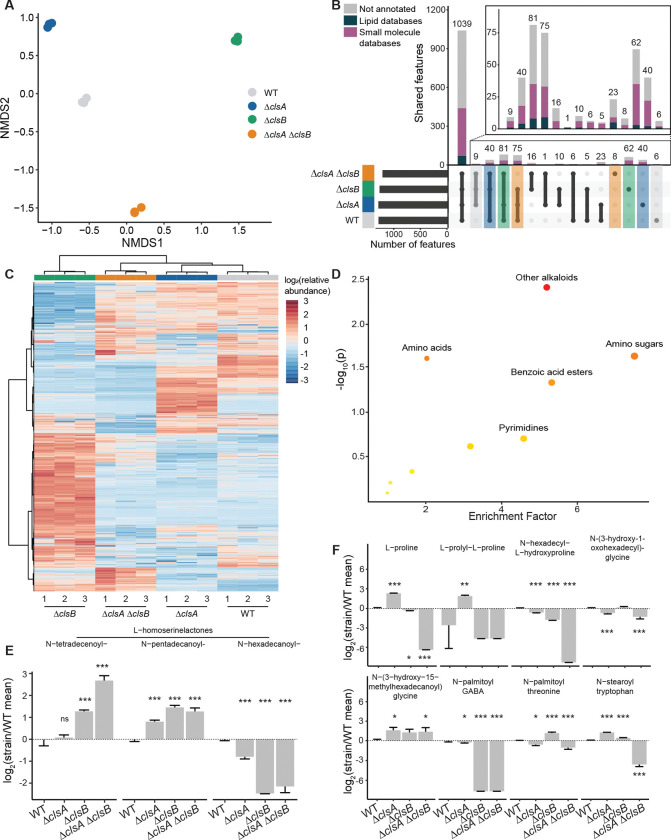
Cardiolipin synthases disrupt metabolic homeostasis. **A.** NMDS of metabolomics data colored by strain. **B.** Upset plot with the main bar plot color coded for the metabolomic features annotation sources (dark green = lipid databases, purple = small molecule databases, grey = not annotated within 5ppm error) and the lower section color coded for metabolomic features only present or absent in each strain (grey = WT, blue = Δ*clsA,* green = Δ*clsB*, orange = Δ*clsA ΔclsB*). Inset is a rescaled axis of metabolites missing in at least one strain. **C.** Heatmap of row normalized abundance of each metabolite in each strain, clustering by metabolomic features on the vertical axis and samples on the horizontal axis using Euclidean distance and Ward clustering (3 technical replicates shown for each strain). **D.** Metabolic pathway analysis with nonlipid sub-categories of *clsAclsB* knockout compared to wildtype (≥3 features/pathway). The x-axis represents the enrichment factor computed from pathway topological analysis on non-lipid species, and the y-axis is the log of p-value obtained from pathway enrichment analysis. The pathways that were most significantly changed are characterized by both a high-log(p) value and high enrichment factor (top right region). **E., F.** Abundance of compounds in enriched pathways for (E) lipid-conjugated homoserine lactones and (F) fatty amides (top enriched pathways) (mean and standard deviation of 3 technical replicates shown; linear regression compared to WT). Across all panels, error bars indicate standard deviation. Unadjusted p-values are shown; *, *p* < 0.05; **, *p* < 0.01; ***, *p* < 0.001.

Since we observed distinct shifts in non-cardiolipin lipids, we hypothesized that these shifts might extend to other *B. fragilis* metabolites under non-stressed conditions. Using LC-tandem mass spectrometry (LC-MS/MS), we found that each strain had unique metabolite profiles, with Δ*clsB* being the most distinct (Figs. 8A–C). Using publicly available databases, 56% of all features were annotated, with 83% of those matches from small molecule databases. Approximately 27% of the metabolomic features were not shared between all the strains, with ~14% of the features only depleted in Δ*clsA*, Δ*clsB*, or Δ*clsA ΔclsB* (Fig. 8B). Notably, we observed that the effect of *clsA* and *clsB* deletion is not additive, with *clsB* deletion showing the most profound alterations on the metabolomic profiles by hierarchical clustering (Fig. 8C).

To determine metabolites that are associated with *cls* deletions, we performed pathway enrichment analysis comparing feature abundances between the WT and double knockout strain (Tables S6, S7). As with the lipidomics analysis above, we identified fatty amides as the most differentially enriched pathway, followed by the fatty esters and sphingolipids (Fig. S8). Several non-lipid classes were also identified, including alkaloids, amino acids, amino sugars, benzoic acid esters, and pyrimidines as the most differentially enriched pathways with high enrichment factors and significant p-values (Fig. 8D). Analysis of amino acids and related compounds identified lipid-conjugated homoserine lactones (HSLs) and fatty amides to be substantially changed in the mutant strains (Figs 8E–F). The lipid-conjugated HSLs, which are small signaling molecules crucial in quorum sensing (79), were mainly associated with *clsB* deletion. In these mutants, levels of long-chain HSLs (e.g., *N*-hexadecanoyl HSL) were reduced, while shorter lipid conjugates (e.g. *N*-tetradecanoyl- and *N*-pentadecanoyl HSL) were enriched. Changes in fatty amides were associated with both *clsA* and *clsB* deletions, with many metabolites showing opposing trends between mutant strains. Levels of several *N*-acyl amides were affected by both *clsA* and *clsB* deletion, including *N*-hexadecyl-L-hydroxyproline, *N*-(3-hydroxy-14-methyldecanoyl) glycine, *N*-palmitoyl threonine, and *N*-stearoyl tryptophan. Some *N*-acyl amides showed gene specific changes such as *N*-(3-hydroxy-1-oxodecanoyl) glycine, which was primarily affected by *clsA* deletion, and *N*-palmitoyl GABA, which was impacted by *clsB* deletion (Fig. 8F). *N*-acyl amides may serve as additional carbon sources and/or signaling molecules between bacteria or to the host, such as through G-protein coupled receptors (GPCRs) (80, 81). Molecular network analysis identified *N*-acyl amides in larger clusters of chemically similar but unannotated metabolites, suggesting that *cls* modifications may lead to larger global metabolomic shifts (Fig. S9A–B). These findings demonstrate that cardiolipin synthases play essential non-redundant roles in maintaining metabolic homeostasis in *B. fragilis* P207.

## DISCUSSION

**Figure 9.**
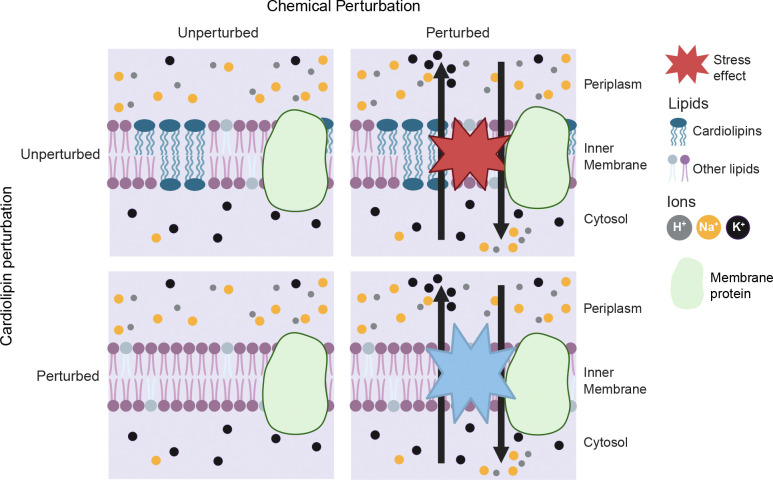
Cardiolipins mitigate the fitness cost of membrane stress in *B. fragilis*. In unperturbed cells, ion gradients are maintained across the inner membrane, with higher extracellular concentrations of H⁺ and Na⁺ and elevated intracellular K⁺ (top left). The outer membrane is generally permeable to these ions due to the presence of porins. In the absence of cardiolipin, *B. fragilis* remodels its membrane lipid composition, which presumably helps to maintain ion gradients (bottom left). Chemical membrane (or membrane ion gradient) perturbation alters *cls* gene expression and ion homeostasis (top right). In the absence of cardiolipins, cells are more susceptible to chemical membrane perturbations (bottom right). The larger blue star indicates the greater fitness cost associated with membrane perturbation in cardiolipin-deficient cells, particularly *clsB* mutants. In contrast, the smaller red star reflects reduced stress susceptibility in cells that retain cardiolipin synthesis.

In this study, we demonstrate that *B. fragilis* P207 has two functionally non-redundant cardiolipin synthases, which we named ClsA and ClsB. Deleting either gene leads to distinct defects in *B. fragilis* fitness under stress, with the loss of *clsB* generally being more detrimental than *clsA* (Fig. 9). We note that *clsA* deletion results in elevated *clsB* expression, which may partially compensate for and minimize the impact of its loss. We present evidence that ClsA and ClsB synthesize cardiolipin species that differ in acyl chain length and unsaturation levels, suggesting that each enzyme has unique substrate specificity and contributes distinct lipids to the membrane. Furthermore, loss of either enzyme has knock-off effects on diverse cellular metabolic processes.

This study is significant for several reasons. First, we offer an approach that integrates metallomics-, lipidomics-, metabolomics-, and classical genetics-based techniques to investigate cardiolipin synthase function. We employ tandem mass spectrometry to assess how *cls* deletion affects the lipid composition of the *B. fragilis* envelope. To our knowledge, previous studies have not leveraged high-resolution mass detection to differentiate the specific lipid species produced by individual Cls proteins (26, 31, 41, 42, 82). While some have used MS/MS to characterize membranes of diverse taxa, including *Bacteroides*, these approaches have not been applied to strains with deletion of individual *cls* genes, limiting the identification of products associated with each enzyme (76, 77, 83–86). By combining genetic and analytical approaches, we distinguish the unique contributions of each Cls to the cardiolipin pool and intracellular ion gradient homeostasis. Second, we provide functional characterization of *cls* genes in the *Bacteroides* taxonomic group, particularly genes from a novel clinical isolate from the IBD gut. As important members of the human gut ecosystem, *Bacteroides* species play key roles in microbiota metabolism and host immune health (46). Understanding how these microbes regulate membrane composition and homeostasis is essential for understanding their fitness in the context of the gut. Future research should continue to explore cardiolipin biosynthesis in these organisms, particularly in *B. thetaiotaomicron*, which possesses an additional *cls* not found in most other *Bacteroides* species.

Due to the high diversity of bacterial *cls* genes, we struggled with the best approach to name the *Bacteroides* group *cls* homologs (34, 82, 87, 88). While new letter codes (e.g. *clsX* or *clsN*) might help distinguish *cls* gene diversity across bacteria, this naming scheme might have become onerous upon characterization of other homologs. We therefore decided on the naming conventions used for *E. coli cls* and other taxa, acknowledging that *cls* homologs across different bacterial phyla may have distinct functional roles (89, 90).

The *B. fragilis cls* genes have some similarity to *E. coli clsA* and *clsB* as the genes from both taxa have strong alignment with cardiolipin synthase domain models. However, at the amino acid sequence level, no clear phylogenetic relationship exists between these homologs. In each taxon, the ClsA homolog is predicted to have two N-terminal transmembrane helices. While these domains in ClsA are thought to be cleaved post-translationally in *E. coli*, it is likely that this does not occur in *Bacillus subtilis* (26, 90). Therefore, the functional consequences of these putative transmembrane domains in *B. fragilis* remain unknown, particularly their role in the cellular localization of these enzymes (90, 91). In addition, across these two taxa, the *cls* expression profiles are distinct. *E. coli* relies almost exclusively on *clsA* during log phase, and expression of *clsB* and *clsC* is largely limited to the stationary phase (26, 31, 41, 42). Under our growth conditions, *B. fragilis* expresses both *cls* genes during log phase, with *clsA* expression further enhanced during stationary phase. To determine if the two *B. fragilis* Cls have overlapping enzymatic activities with the Cls of these other taxa, comparative biochemical analysis of the lipid products produced by the Cls homologs of each species is needed.

Maintaining ion gradients across membranes is essential for bacterial survival, as these gradients provide a reservoir of potential energy. The loss of cardiolipin biosynthesis under non-stressed conditions largely does not affect ion homeostasis, as seen in our *B. fragilis* metallomic studies. Bacteria generally tightly regulate intracellular K^+^ content due to its physiological importance, with some adjusting it by several fold depending on extracellular conditions (24, 92). For example, *B. subtilis* and *E. coli* both increase intracellular K^+^ levels in response to elevated osmolarity (93, 94). However, *S. aureus* does not modulate K^+^ content in this manner (95). While one might expect ion gradient perturbations given previous work showing improved membrane stability with the presence of cardiolipin (88, 96, 97), the resilience of intracellular K^+^ and Na^+^ levels in the absence of cardiolipin suggests that otherwise unperturbed cells maintain membrane integrity. Even if loss of cardiolipin changed the inherent membrane permeability of either K^+^ or Na^+^, our data indicate that *B. fragilis* leverages membrane remodeling and several compensatory ion shuttling systems to achieve and maintain intracellular homeostasis (Fig. 5C–D) (24, 92, 98–103).

While *B. fragilis clsA* is not essential and may even diminish fitness during selective ion permeabilization, both *cls* genes are beneficial when the bacterium faces broader membrane stress conditions. Although unlikely, the absence of ClsA-associated cardiolipins in the Δ*clsA* strain may provide stress resistance by impairing ionophore translocation across the membrane (59, 104, 105). Our favored hypothesis is that Δ*clsA*-dependent membrane remodeling enhances resilience to ion gradient loss, possibly through altered membrane protein or lipid function. Beyond general effects like membrane fluidity, cardiolipins can influence bacterial physiology through multiple pathways. For example, some proteins that influence cell shape and cell division, such as the actin homolog MreB, rely on cardiolipin for proper folding at the membrane (106, 107). Interactions of cardiolipin with membrane-bound or membrane-associated cell division/shape proteins may also underlie our observation that the loss of cardiolipin biosynthesis results in *B. fragilis* cell shape defects (60–66). The cls-specific cell size effects may be shaped by asymmetrical distribution of cardiolipin species across inner and outer leaflets of the inner membrane as well as inner leaflet of the outer membrane (16, 108–110).

The catalytic products of *B. fragilis* ClsA and ClsB, as inferred from our lipidomics data, have distinct acyl chain lengths and unsaturation levels. In response to environmental changes, bacteria can adjust these lipid properties, which are key factors influencing membrane fluidity and permeability (111). For example, under conditions that increase membrane rigidity, such as lower temperature or high pressure, microbes increase the abundance of highly unsaturated fatty acids to restore membrane fluidity (112, 113). Exposure to membrane destabilizing agents, such as alcohols, can result in decreased acyl chain unsaturation (114). Exposure to phenols, which can also disrupt membranes, cause some anaerobes to increase their membrane fluidity by incorporating fatty acids with trans-double bonds (115). Although *B. fragilis* apparently lacks enzymes required for this particular mechanism of introducing double bonds, phylogenetically diverse bacteria have other regulatory mechanisms for the introduction of double bonds into acyl chains already present in the membrane (116, 117). Having two unique pools of cardiolipins with corresponding membrane lipids that respond to cardiolipin fluctuations, such as hexosylceramide for ClsA and *N*-acyl glycylserine for ClsB, would provide bacteria like *B. fragilis* with greater adaptability in the face of diverse selective pressures in the gut.

The distinct metabolomic alterations observed in *B. fragilis* ClsA and ClsB mutants underscore the non-redundant and critical roles of cardiolipin synthases in bacterial metabolic homeostasis. Our results demonstrate that loss of either ClsA or ClsB leads to unique changes in the abundance of key lipid and non-lipid metabolites. These changes likely reflect the broader disruptions in membrane composition and cellular signaling, as well as shifts in central metabolic pathways. Importantly, the non-additive nature of the double mutant phenotype suggests that ClsA and ClsB may influence partially overlapping yet distinct biosynthetic networks. The pronounced depletion and enrichment of specific metabolites, such as lipid-conjugated HSLs and *N*-acyl amides, further indicates that cardiolipin synthase activity influences both membrane structure and intercellular communication. Given the essential role of membrane integrity and metabolic adaptability in bacterial survival within the gut environment, these findings suggest that ClsA and ClsB are pivotal for the ecological fitness of *B. fragilis*.

In summary, *B. fragilis* relies on cardiolipin synthases to maintain barrier integrity against diverse stresses that it encounters in the gut, such as bile acids and osmotic variation. However, much remains to be discovered regarding *B. fragilis* Cls-mediated physiology, including protein domain function, cellular localization, and the mechanism by which each enzyme produces distinct cardiolipins. Furthermore, the overlapping, yet possibly distinct, functional roles that this diverse enzyme class plays across the prokaryotic tree of life is ripe with potential. The data we present here serve as a foundation for a deeper understanding of *B. fragilis* physiology specifically as well as that of the Bacteroidota generally, potentially leading to the engineering of probiotic gut species with improved gut fitness and therapeutic outcomes.

## METHODS (see supplemental methods for additional detail)

### Bacterial Growth and Strains

#### Strain generation

*E. coli* strains were grown aerobically at 37° C in Miller LB broth. As appropriate, E. coli cultures were supplemented with filter-sterilized 300 μM diaminopimelic acid (Sigma-Aldrich) to grow auxotrophic strains and/or 100 μg/ml carbenicillin to select for plasmid containing cells. All *B. fragilis* strains were grown in supplemented brain heart infusion (BHIS) medium. As appropriate, cultures were supplemented with 5 μg/mL erythromycin for vector selection, 100 ng/mL anhydrotetracycline (aTC) for inducible ssbfe1 counter-selection (118) and 50 ng/mL aTC to induce expression from aTC-inducible complementation vectors (118). *B. fragilis* strains were manipulated aerobically and grown at 37°C in an anaerobic chamber (Coy Laboratory Products) filled with 5% CO_2_, 2.5% hydrogen and 92.5% nitrogen.

Mutant strains were generated by allelic exchange using modified vectors described previously (118). For conjugation into *B. fragilis* P207, plasmids were transformed into the donor *E. coli* strain WM3064. Allele exchange was conducted as previously described (118). The Δ*clsA* mutant (lacking ptos_000612) was complemented by integration of an aTC-inducible *clsA* construct. The Δ*clsB* mutant (lacking ptos_003215) was complemented by integration of *clsB* under the control of a constitutive *Bacteroides* promoter (P_*rpoD*_, bt_1311*/*BT_RS06635). Complementation constructs were integrated into the chromosome at the *att* site using an intN1 integrase (119). Primers, vectors, and strains are listed in Tables S1.

### Cls conservation analysis

#### Alignment.

The *B. fragilis* P207 ClsA and ClsB gene and protein sequences, as well as *E. coli* K-12 MG1655 (NC_000913) ClsA, ClsB, and ClsC were aligned using default settings of the Geneious alignment algorithm (Geneious Prime 2024) (51, 52).

#### DeepTMHMM.

The ClsA and ClsB amino acid sequences from *B. fragilis* P207 and *E. coli* K-12 MG1655 were uploaded to DeepTMHMM (v. 1.0.42, https://dtu.biolib.com/DeepTMHMM) to predict transmembrane domains (120).

#### Inter-Phylum Tree.

Representative taxa were selected from the top five phyla in the human gut (metadata listed in Table S2) (121, 122). Annotated *cls* genes were obtained from genomes on NCBI and protein aligned in Geneious. One genome per species was used, aside from the two *B. fragilis* strains. The phylogenetic tree was reconstructed using neighbor-end joining via Geneious and then visualized using R.

#### *Intra-*Bacteroides *Tree.*

TIGR04265 domains in *B. fragilis* ClsA and ClsB were identified using the conserved domain database of NCBI (123). *Bacteroides* protein sequences containing this domain were obtained by querying the Interpro database. Alignment, tree reconstruction, and tree visualization were performed as with the inter-phylum tree, except that *E. coli* K-12 MG1655 ClsA was specified as an outgroup.

#### Genomic Neighborhoods.

WebFlags2 was used to query genomic neighborhoods around *B. fragilis* P207 *clsA* and *clsB* genes (124).

### Growth curve assays

Stationary phase *B. fragilis* strain cultures were back-diluted to an optical density at 600 nm (OD_600_) of 0.05 and grown to OD_600_ 0.3 in the presence of 50 ng/mL aTC inducer. Each strain was then diluted again to 0.025–0.05 for growth curves in a 96-well plate format. OD_600_ was measured with a Tecan Infinite M Nano plate reader (Tecan, Männedorf, Switzerland) for 24 h. Three biological replicates, each in technical triplicate, were performed for each growth condition.

To expose cells to stress conditions, BHIS medium was prepared as above but modified. For pH assays, cells were pelleted and then resuspended in aTC-containing BHIS medium adjusted to the appropriate pH. For deoxycholate and SDS assays, 0.01% (w/v) deoxycholate (Sigma) or 0.002% (w/v) SDS (Lab Scientific) was added to cultures from filter-sterilized 1% (w/v) stocks dissolved in UltraPure water. For Na^+^ and K^+^ assays, NaCl (Fisher Scientific) and KCl (Fisher Scientific) were dissolved to a 4× concentration of each cation in BHIS medium and then filter sterilized before being added to cells to yield the final concentration of cells and cation (i.e., 300 mM). To determine how much additional NaCl or KCl to add, the average Na^+^ and K^+^ content of our BHIS medium was determined using ICP-MS (see below for methods), yielding 130 mM for Na^+^ and 33 mM for K^+^ (Table S4). For ionophore assays, a concentrated ionophore (monensin A or nigericin; Sigma-Aldrich) was added to cultures to yield final concentrations of 0.8 μg/mL monensin A or 0.1 μg/mL nigericin, with equivalent volumes of ethanol added to non-treated conditions as a vehicle control. We calculated maximum OD600 and doubling time using R (ipolygrowth; see supplemental methods) (125).

### Microscopy

*B. fragilis* P207 cell cultures were grown overnight under antibiotic selection and then back-diluted into medium containing inducer (aTC) without antibiotic selection to reach log-phase growth. All cultures were removed from the 37°C incubator but were maintained under anaerobic conditions, with only two or three cultures removed for microscopy at a time. Cells were visualized by phase-contrast microscopy on a Leica DMI6000 B inverted microscope with a Hamamatsu ORCA-R2 10600 camera with a 63× Plan Apo objective (126). Each strain was visualized on four different days to generate four biological replicates. The two images per replicate from each strain/day were chosen for analysis. Chosen images were processed using the MicrobeJ plugin (v. 5.13I) cell size analysis software in Fiji (v. 1.54k) (127). Called cells were manually curated and resulting cell lengths and widths were exported and analyzed in R to calculate cell volume and Epanechnikov kernel densities.

### Gene expression assays

#### Stress condition exposure:

For comparison of *cls* gene expression at log and stationary phases, *B. fragilis* WT, Δ*clsA* and Δ*clsB* strain log phase cultures were back diluted to an OD_600_ of 0.05 without inducer. Two and 24 h later, cell samples were pelleted and resuspended in 1 mL of Trizol (Invitrogen). For acute toxicity assays, back-diluted WT *B. fragilis* cultures that reached OD_600_ ~0.3–0.5 were exposed to a specified condition (i.e., pH, SDS, deoxycholate, K^+^, monensin A, or nigericin, as described for the growth assays) and incubated for 20 min before cell sample collection.

#### RNA extraction:

RNA was extracted as described previously (51). RNeasy Extraction Kit (Qiagen) was used to treat samples with Turbo DNase (Invitrogen) using the manufacturers protocol.

#### RT-qPCR:

Samples were run in technical triplicate. *Ct* values were determined using a QuantStudio5 (Applied Biosystems) instrument and a Luna One-Step RT-qPCR kit (NEB) for target genes in each sample (51). Using *B. fragilis dnaN* (ptos_002600, encoding DNA polymerase subunit beta) as a normalization gene.

### Inductively coupled plasma mass spectrometry

Log phase cells were removed from the anaerobic chamber for sample collection. If exposed to a stress, cells were incubated for 20 min aerobically at room temperature with either 0.01% (w/v) deoxycholate, 0.8 μg/mL monensin A or 0.1 μg/mL nigericin. Cultures were spiked with gadolinium-DOTA (Gd-DOTA, Macrocyclics) to a final concentration of 40 μM. Washing cells, even with the BHIS medium cells were grown in, perturbs intracellular concentrations of small alkali metals like Na^+^ and K^+^ making quantification difficult (Fig. S5A). Using a Gd-DOTA spike enabled us to remove washing steps in the ICP-MS sample preparation process (128, 129).

For the wash experiment, wash condition cells were washed twice with either 250 mM sucrose or BHIS media. Then, unwashed cells (i.e., no previous centrifugation) and resuspended washed cells were transferred to a metal-free tube, given a Gd-DOTA spike to 40 μM, and then pelleted. For *cls* and treatment comparison experiments, cells were unwashed.

Weighed cell pellets and supernatant were dried overnight, then digested using 70% HNO_2_ acid, before diluting to 3% nitric acid. All standards, blanks, and ICP-MS samples were prepared using ultra-trace metal grade nitric acid (70%, Fisher), ultrapure water (Millipore), metal free polypropylene conical tubes (15 and 50 mL, Labcon), and trace metal grade pipette tips (Labcon). All solutions were weighed for accurate elemental determination using the XSR205 DU semi-analytical balance (Mettler Toledo).

Samples were analyzed using an Agilent 8900 Triple Quadrupole ICP-MS (Agilent Technologies). To prepare ICP-MS standards, the multi element standard IV-65024 (Inorganic Ventures) was diluted with 3% (v/v) nitric acid in ultrapure water. Internal standardization was accomplished inline using a 200 ppb internal standard solution in 3% (v/v) nitric acid in ultrapure water (IV-ICPMS-71D, Inorganic Ventures). The isotopes selected for analysis were ^23^Na, ^31^P, ^32^S, ^39^K and ^157^Gd with ^45^Sc, ^89^Y, ^115^In, and ^159^Tb used for internal standardization. For accurate quantification of ^31^P and ^32^S, these isotopes were measured in the oxygen mode. To quantify intracellular elemental concentration, the average strain-specific cell volume was determined and then the molar concentration calculated for each element from the number of atoms per cell. The ΔmM concentrations were calculated by subtracting the concentration of an element in the medium from that in the intracellular space.

### Lipidomics assays

WT, Δ*clsA*, Δ*clsB*, and Δ*clsA ΔclsB* strains were backdiluted, grown to OD_600_ 0.7, and then submitted on dry ice to the University of Tennessee-Knoxville Biological and Small Molecule Mass Spectrometry Core (BSMMSC; RRID: SCR_021368) for targeted lipidomic analysis of membrane lipids. Lipids were extracted as previously described (see supplemental methods for details) (130, 131). The lipidomic analyses were performed using a previously validated method (131) on an ultra high performance liquid chromatography system coupled to a high resolution mass spectrometer (UHPLC-HRMS). The chromatographic separations were carried out using Vanquish Horizon LC system (Thermo Scientific) and reversed phase separations as described previously (131). The eluent was introduced to an Exploris 120 mass spectrometer (Thermo Scientific) via electrospray ionization for high resolution analysis. Following the analysis, the lipids were annotated and the peak area integrated using MS-DIAL (132–135). Peak area for a metabolite was first normalized to the optical density of a sample, then relative lipid abundance was determined by normalizing to the total peak area of all lipids detected in each UHPLC–MS/HRMS mode (i.e., positive versus negative). Elastic net regression performed using the glmnet R package (136, 137).

### Metabolomic assays

WT, Δ*clsA*, Δ*clsB*, and Δ*clsA ΔclsB* strains were grown in 50mL BHIS medium and metabolites were extracted with ethyl acetate (see supplemental methods for details). Untargeted metabolomics data was acquired using a Bruker timsTOF Pro2 (Bruker-Daltonics, Billerica, MA, USA) coupled to an Agilent 1290 Infinity II Bio UHPLC (Agilent, Santa Clara, CA, USA). The chromatographic separations were carried out using an Acquity UPLC HSST3 column (2.1 × 150mm, 1.8 μM) (see supplemental methods for details). Following data acquisition, mass spectrometry (MS) data were pre-processed using Bruker MetaboScape^®^ version 9.0.1 (Bruker-Daltonics, Billerica, MA, USA) and filtered using mpactR (see supplemental methods for details) (138). Peak intensity for each metabolite was normalized to the total peak intensity of all features detected in the sample. Molecular networking was generated as previously described (139) using GNPS2: Global Natural Products Social Molecular Networking 2 (http://gnps2.org/). A minimum cosine similarity score of 0.7 with at least six matching peaks, parent mass tolerance of 2 Da, and fragment ion tolerance 0.5 Da were selected to generate consensus spectra. Files were imported into CytoScape 3.10.3 and nodes were arranged with yFiles organic layout plugin (140). Pathway analysis was performed with MetaboAnalyst 6.0 functional analysis [LC-MS] module (Tables S6, S7) (141). A formatted peak intensity table was uploaded to the web server (https://metaboanalyst.ca), then normalized by sum and log transformed. Default setting of 5.0 ppm mass tolerance, mummichog algorithm v2.0 with p-value cutoff of 0.0005, against the lipid and non-lipid pathway libraries with at least 3 entries in each pathway. Metabolites from enriched pathways were plotted using R (v4.5.0).

### Data analysis and reproducibility

All data analyses were performed using R (v4.3.3 unless otherwise specified) and several packages (125, 136, 137, 142–164). Code used and original datasets for all data analysis and figure generation, except for the WebFlags neighborhoods, is published on GitHub (https://github.com/mschnizlein/bfrag_cardiolipin). Lipidomics data alongside appropriate metadata were deposited on MetaboLights (MTBLS11891 www.ebi.ac.uk/metabolights/MTBLS11891) (165). Untargeted metabolomics were deposited to MassIVE (https://massive.ucsd.edu) (deposit in progress at time of submission).

## Supplementary Material

Supplement 1

Supplement 2

Supplement 3

Supplement 4

Supplement 5

Supplement 6

Supplement 7

Supplement 8

Supplement 9

## References

[1] Schnizlein MK, Young VB. 2022. Capturing the environment of the *Clostridioides difficile* infection cycle. Nature Reviews Gastroenterology & Hepatology 19:508–520. 35468953 10.1038/s41575-022-00610-0

[2] Shortt C, Hasselwander O, Meynier A, Nauta A, Fernández EN, Putz P, Rowland I, Swann J, Türk J, Vermeiren J, Antoine J-M. 2018. Systematic review of the effects of the intestinal microbiota on selected nutrients and non-nutrients. European Journal of Nutrition 57:25–49. 29086061 10.1007/s00394-017-1546-4 PMC5847024

[3] Kern L, Abdeen SK, Kolodziejczyk AA, Elinav E. 2021. Commensal inter-bacterial interactions shaping the microbiota. Current Opinion in Microbiology 63:158–171. 34365152 10.1016/j.mib.2021.07.011

[4] Runge S, Rosshart SP. 2021. The mammalian metaorganism: A holistic view on how microbes of all kingdoms and niches shape local and systemic immunity. Frontiers in Immunology 12. 10.3389/fimmu.2021.702378 PMC8278200 34276696

[5] He X, McLean JS, Guo L, Lux R, Shi W. 2014. The social structure of microbial community involved in colonization resistance. ISME J 8:564–574. 24088624 10.1038/ismej.2013.172 PMC3930314

[6] Wennerström H, Oliveberg M. 2022. On the osmotic pressure of cells. QRB Discovery 3:e12. 37529285 10.1017/qrd.2022.3 PMC10392628

[7] Denyer SP, Maillard JY. 2002. Cellular impermeability and uptake of biocides and antibiotics in Gram-negative bacteria. Journal of Applied Microbiology 92:35S-45S. 12000611

[8] Zhang Y-M, Rock CO. 2008. Membrane lipid homeostasis in bacteria. Nature Reviews Microbiology 6:222–233. 18264115 10.1038/nrmicro1839

[9] Strahl H, Errington J. 2017. Bacterial membranes: Structure, domains, and function. Annual Review of Microbiology 71:519–538. 10.1146/annurev-micro-102215-095630 28697671

[10] Sohlenkamp C, Geiger O. 2015. Bacterial membrane lipids: Diversity in structures and pathways. FEMS Microbiology Reviews 40:133–159. 25862689 10.1093/femsre/fuv008

[11] Blevins MS, James VK, Herrera CM, Purcell AB, Trent MS, Brodbelt JS. 2020. Unsaturation elements and other modifications of phospholipids in bacteria: New insight from ultraviolet photodissociation mass spectrometry. Analytical Chemistry 92:9146–9155. 32479092 10.1021/acs.analchem.0c01449 PMC7384744

[12] Roy H, Dare K, Ibba M. 2009. Adaptation of the bacterial membrane to changing environments using aminoacylated phospholipids. Molecular Microbiology 71:547–550. 19054327 10.1111/j.1365-2958.2008.06563.x PMC2774118

[13] Cox E, Michalak A, Pagentine S, Seaton P, Pokorny A. 2014. Lysylated phospholipids stabilize models of bacterial lipid bilayers and protect against antimicrobial peptides. Biochimica et Biophysica Acta (BBA) - Biomembranes 1838:2198–2204. 24780374 10.1016/j.bbamem.2014.04.018 PMC4118599

[14] Donohue TJ, Cain BD, Kaplan S. 1982. Alterations in the phospholipid composition of *Rhodopseudomonas sphaeroides* and other bacteria induced by Tris. Journal of Bacteriology 152:595–606. 6982264 10.1128/jb.152.2.595-606.1982 PMC221506

[15] Lin T-Y, Weibel DB. 2016. Organization and function of anionic phospholipids in bacteria. Applied Microbiology and Biotechnology 100:4255–4267. 27026177 10.1007/s00253-016-7468-x

[16] Bogdanov M, Pyrshev K, Yesylevskyy S, Ryabichko S, Boiko V, Ivanchenko P, Kiyamova R, Guan Z, Ramseyer C, Dowhan W. 2020. Phospholipid distribution in the cytoplasmic membrane of Gram-negative bacteria is highly asymmetric, dynamic, and cell shape-dependent. Science Advances 6:eaaz6333. 32537497 10.1126/sciadv.aaz6333 PMC7269648

[17] Poolman B. 2023. Physicochemical homeostasis in bacteria. FEMS Microbiology Reviews 47. 10.1093/femsre/fuad033 PMC10368375 37336577

[18] Zharova TV, Grivennikova VG, Borisov VB. 2023. F_1_·F_o_ ATP synthase/ATPase: Contemporary view on unidirectional catalysis. International Journal of Molecular Sciences 24:5417. 36982498 10.3390/ijms24065417 PMC10049701

[19] Haddock BA, Jones CW. 1977. Bacterial respiration. Bacteriol Rev 41:47–99. 140652 10.1128/br.41.1.47-99.1977 PMC413996

[20] Lu Z, Imlay JA. 2021. When anaerobes encounter oxygen: Mechanisms of oxygen toxicity, tolerance and defence. Nature Reviews Microbiology 19:774–785. 34183820 10.1038/s41579-021-00583-y PMC9191689

[21] Ito T, Gallegos R, Matano LM, Butler NL, Hantman N, Kaili M, Coyne MJ, Comstock LE, Malamy MH, Barquera B. 2020. Genetic and biochemical analysis of anaerobic respiration in *Bacteroides fragilis* and its importance *in vivo*. mBio 11:e03238–19. 32019804 10.1128/mBio.03238-19 PMC7002350

[22] Butler NL, Ito T, Foreman S, Morgan JE, Zagorevsky D, Malamy MH, Comstock LE, Barquera B. 2023. *Bacteroides fragilis* maintains concurrent capability for anaerobic and nanaerobic respiration. Journal of Bacteriology 205:e00389–22. 36475831 10.1128/jb.00389-22 PMC9879120

[23] Beagle SD, Lockless SW. 2021. Unappreciated roles for K^+^ channels in bacterial physiology. Trends in Microbiology 29:942–950. 33288383 10.1016/j.tim.2020.11.005 PMC9159956

[24] Stautz J, Hellmich Y, Fuss MF, Silberberg JM, Devlin JR, Stockbridge RB, Hänelt I. 2021. Molecular mechanisms for bacterial potassium homeostasis. Journal of Molecular Biology 433:166968. 33798529 10.1016/j.jmb.2021.166968 PMC9041122

[25] Schlame M. 2008. *Thematic Review Series: Glycerolipids.* Cardiolipin synthesis for the assembly of bacterial and mitochondrial membranes. Journal of Lipid Research 49:1607–1620. 18077827 10.1194/jlr.R700018-JLR200 PMC2444000

[26] Tropp BE. 1997. Cardiolipin synthase from *Escherichia coli*. Biochimica et Biophysica Acta (BBA) - Lipids and Lipid Metabolism 1348:192–200. 9370333 10.1016/s0005-2760(97)00100-8

[27] Pinkas D, Fišer R, Kozlík P, Dolejšová T, Hryzáková K, Konopásek I, Mikušová G. 2020. *Bacillus subtilis* cardiolipin protects its own membrane against surfactin-induced permeabilization. Biochimica et Biophysica Acta (BBA) - Biomembranes 1862:183405. 32593615 10.1016/j.bbamem.2020.183405

[28] Chen Q-P, Li Q-T. 2001. Effect of cardiolipin on proton permeability of phospholipid liposomes: The role of hydration at the lipid–water interface. Archives of Biochemistry and Biophysics 389:201–206. 11339809 10.1006/abbi.2001.2319

[29] Domitin S, Puff N, Pilot-Storck F, Tiret L, Joubert F. 2025. Role of cardiolipin in proton transmembrane flux and localization. Biophysical Journal 124:408–416. 39674891 10.1016/j.bpj.2024.12.015 PMC11788487

[30] Shibata A, Ikawa K, Shimooka T, Terada H. 1994. Significant stabilization of the phosphatidylcholine bilayer structure by incorporation of small amounts of cardiolipin. Biochimica et Biophysica Acta (BBA) - Biomembranes 1192:71–78. 8204653 10.1016/0005-2736(94)90144-9

[31] Jeucken A, Helms JB, Brouwers JF. 2018. Cardiolipin synthases of *Escherichia coli* have phospholipid class specific phospholipase D activity dependent on endogenous and foreign phospholipids. Biochimica et Biophysica Acta (BBA) - Molecular and Cell Biology of Lipids 1863:1345–1353. 29933046 10.1016/j.bbalip.2018.06.017

[32] Domitin S, Puff N, Pilot-Storck F, Tiret L, Joubert F. 2025. Role of cardiolipin in proton transmembrane flux and localization. Biophys J 124:408–416. 39674891 10.1016/j.bpj.2024.12.015 PMC11788487

[33] Shibata A, Ikawa K, Shimooka T, Terada H. 1994. Significant stabilization of the phosphatidylcholine bilayer structure by incorporation of small amounts of cardiolipin. Biochim Biophys Acta 1192:71–8. 8204653 10.1016/0005-2736(94)90144-9

[34] Yeo W-S, Dyzenhaus S, Torres VJ, Brinsmade SR, Bae T. 2023. Regulation of bacterial two-component systems by cardiolipin. Infection and Immunity 91:e00046–23. 36975788 10.1128/iai.00046-23 PMC10112254

[35] Ryabichko S, Ferreira VdM, Vitrac H, Kiyamova R, Dowhan W, Bogdanov M. 2020. Cardiolipin is required *in vivo* for the stability of bacterial translocon and optimal membrane protein translocation and insertion. Scientific Reports 10:6296. 32286407 10.1038/s41598-020-63280-5 PMC7156725

[36] Musatov A, Sedlák E. 2017. Role of cardiolipin in stability of integral membrane proteins. Biochimie 142:102–111. 28842204 10.1016/j.biochi.2017.08.013

[37] Hong Y, Muenzner J, Grimm SK, Pletneva EV. 2012. Origin of the conformational heterogeneity of cardiolipin-bound cytochrome c. Journal of the American Chemical Society 134:18713–18723. 23066867 10.1021/ja307426k PMC3507619

[38] Arias-Cartin R, Grimaldi S, Arnoux P, Guigliarelli B, Magalon A. 2012. Cardiolipin binding in bacterial respiratory complexes: Structural and functional implications. Biochimica et Biophysica Acta (BBA) - Bioenergetics 1817:1937–1949. 22561115 10.1016/j.bbabio.2012.04.005

[39] Planas-Iglesias J, Dwarakanath H, Mohammadyani D, Yanamala N, Kagan Valerian E, Klein-Seetharaman J. 2015. Cardiolipin interactions with proteins. Biophysical Journal 109:1282–1294. 26300339 10.1016/j.bpj.2015.07.034 PMC4576322

[40] Hirschberg CB, Kennedy EP. 1972. Mechanism of the enzymatic synthesis of cardiolipin in *Escherichia coli*. Proceedings of the National Academy of Sciences 69:648–651. 10.1073/pnas.69.3.648 PMC426527 4551982

[41] Tan BK, Bogdanov M, Zhao J, Dowhan W, Raetz CR, Guan Z. 2012. Discovery of a cardiolipin synthase utilizing phosphatidylethanolamine and phosphatidylglycerol as substrates. Proceedings of the National Academy of Sciences 109:16504–16509. 10.1073/pnas.1212797109 PMC3478633 22988102

[42] Guo D, Tropp BE. 2000. A second *Escherichia coli* protein with CL synthase activity. Biochimica et Biophysica Acta (BBA) - Molecular and Cell Biology of Lipids 1483:263–274. 10634942 10.1016/s1388-1981(99)00193-6

[43] Li C, Tan BK, Zhao J, Guan Z. 2016. *In Vivo* and *in Vitro* synthesis of phosphatidylglycerol by an *Escherichia coli* cardiolipin synthase. Journal of Biological Chemistry 291:25144–25153. 27760827 10.1074/jbc.M116.762070 PMC5122781

[44] Homma H, Nojima S. 1982. Synthesis of various phospholipids from 2-acyl lysophospholipids by *Escherichia coli* extract. The Journal of Biochemistry 91:1103–1110. 7047508 10.1093/oxfordjournals.jbchem.a133792

[45] Patrick S. 2022. A tale of two habitats: *Bacteroides fragilis*, a lethal pathogen and resident in the human gastrointestinal microbiome. Microbiology 168. 10.1099/mic.0.001156 35404220

[46] Wexler HM. 2007. *Bacteroides*: The good, the bad, and the nitty-gritty. Clin Microbiol Rev 20:593–621. 17934076 10.1128/CMR.00008-07 PMC2176045

[47] Tally FP, Stewart PR, Sutter VL, Rosenblatt JE. 1975. Oxygen tolerance of fresh clinical anaerobic bacteria. J Clin Microbiol 1:161–4. 1176601 10.1128/jcm.1.2.161-164.1975 PMC275000

[48] Valguarnera E, Wardenburg JB. 2020. Good Gone Bad: One Toxin Away From Disease for Bacteroides fragilis. J Mol Biol 432:765–785. 31857085 10.1016/j.jmb.2019.12.003

[49] Scott N, Whittle E, Jeraldo P, Chia N. 2022. A systemic review of the role of enterotoxic *Bacteroides fragilis* in colorectal cancer. Neoplasia 29:100797. 35461079 10.1016/j.neo.2022.100797 PMC9046963

[50] Sun F, Zhang Q, Zhao J, Zhang H, Zhai Q, Chen W. 2019. A potential species of next-generation probiotics? The dark and light sides of *Bacteroides fragilis* in health. Food Res Int 126:108590. 31732047 10.1016/j.foodres.2019.108590

[51] Fiebig A, Schnizlein MK, Pena-Rivera S, Trigodet F, Dubey AA, Hennessy MK, Basu A, Pott S, Dalal S, Rubin D, Sogin ML, Eren AM, Chang EB, Crosson S. 2024. Bile acid fitness determinants of a Bacteroides fragilis isolate from a human pouchitis patient. mBio 15:e02830–23. 38063424 10.1128/mbio.02830-23 PMC10790697

[52] Vineis JH, Ringus DL, Morrison HG, Delmont TO, Dalal S, Raffals LH, Antonopoulos DA, Rubin DT, Eren AM, Chang EB, Sogin ML. 2016. Patient-specific *Bacteroides* genome variants in pouchitis. mBio 7:e01713–16. 27935837 10.1128/mBio.01713-16 PMC5111406

[53] Akiyama S, Rai V, Rubin DT. 2021. Pouchitis in inflammatory bowel disease: a review of diagnosis, prognosis, and treatment. Intest Res 19:1–11. 33138344 10.5217/ir.2020.00047 PMC7873408

[54] Sitaraman R. 2013. Phospholipid catabolism by gut microbiota and the risk of cardiovascular disease. J Med Microbiol 62:948–950. 23518648 10.1099/jmm.0.053587-0 PMC4080731

[55] Romantsov T, Gonzalez K, Sahtout N, Culham DE, Coumoundouros C, Garner J, Kerr CH, Chang L, Turner RJ, Wood JM. 2018. Cardiolipin synthase A colocalizes with cardiolipin and osmosensing transporter ProP at the poles of *Escherichia coli* cells. Molecular Microbiology 107:623–638. 29280215 10.1111/mmi.13904

[56] Quigley BR, Tropp BE. 2009. *E. coli* cardiolipin synthase: Function of N-terminal conserved residues. Biochimica et Biophysica Acta (BBA) - Biomembranes 1788:2107–2113. 19341704 10.1016/j.bbamem.2009.03.016

[57] Antonenko YN, Yaguzhinsky LS. 1988. The ion selectivity of nonelectrogenic ionophores measured on a bilayer lipid membrane: Nigericin, monensin, A23187 and lasalocid A. Biochim Biophys Acta 938:125–30. 19927398 10.1016/0005-2736(88)90151-4

[58] Li J, Ding L, Wang E, Dong S. 1996. The ion selectivity of monensin incorporated phospholipid/alkanethiol bilayers. Journal of Electroanalytical Chemistry 414:17–21.

[59] Łowicki D, Huczyński A. 2013. Structure and antimicrobial properties of monensin A and its derivatives: Summary of the achievements. BioMed Research International 2013:742149. 23509771 10.1155/2013/742149 PMC3586448

[60] Lin T-Y, Gross William S, Auer George K, Weibel Douglas B. 2019. Cardiolipin alters *Rhodobacter sphaeroides* cell shape by affecting peptidoglycan precursor biosynthesis. mBio 10. 10.1128/mBio.02401-18 PMC6381277 30782656

[61] Elías-Wolff F, Lindén M, Lyubartsev AP, Brandt EG. 2019. Curvature sensing by cardiolipin in simulated buckled membranes. Soft Matter 15:792–802. 30644502 10.1039/c8sm02133c

[62] Luchini A, Cavasso D, Radulescu A, D’Errico G, Paduano L, Vitiello G. 2021. Structural organization of cardiolipin-containing vesicles as models of the bacterial cytoplasmic membrane. Langmuir 37:8508–8516. 34213914 10.1021/acs.langmuir.1c00981

[63] Labajová N, Baranova N, Jurásek M, Vácha R, Loose M, Barák I. 2021. Cardiolipin-containing lipid membranes attract the bacterial cell division protein DivIVA. International Journal of Molecular Sciences 22:8350. 34361115 10.3390/ijms22158350 PMC8348161

[64] Renner LD, Weibel DB. 2011. Cardiolipin microdomains localize to negatively curved regions of *Escherichia coli* membranes. Proceedings of the National Academy of Sciences 108:6264–6269. 10.1073/pnas.1015757108 PMC3076878 21444798

[65] Huang KC, Mukhopadhyay R, Wingreen NS. 2006. A curvature-mediated mechanism for localization of lipids to bacterial poles. PLOS Computational Biology 2:e151. 17096591 10.1371/journal.pcbi.0020151 PMC1635540

[66] Bailey BWA, Stansfeld PJ. 2024. The bacterial cytoskeleton alters the cell membrane in molecular dynamics simulations. Biophysical Journal 123:374a.38196191

[67] Rowlett VW, Mallampalli VKPS, Karlstaedt A, Dowhan W, Taegtmeyer, Margolin W, Vitrac H. 2017. Impact of membrane phospholipid alterations in *Escherichia coli* on cellular function and bacterial stress adaptation. Journal of Bacteriology 199:10.1128/jb.00849-16. PMC5472821 28439040

[68] Duncan AL, Robinson AJ, Walker JE. 2016. Cardiolipin binds selectively but transiently to conserved lysine residues in the rotor of metazoan ATP synthases. Proc Natl Acad Sci U S A 113:8687–92. 27382158 10.1073/pnas.1608396113 PMC4978264

[69] Mehdipour AR, Hummer G. 2016. Cardiolipin puts the seal on ATP synthase. Proc Natl Acad Sci U S A 113:8568–70. 27439859 10.1073/pnas.1609806113 PMC4978257

[70] Shapiro JS, Chang H-C, Tatekoshi Y, Zhao Z, Waxali ZS, Hong BJ, Chen H, Geier JA, Bartom ET, De Jesus A, Nejad FK, Mahmoodzadeh A, Sato T, Ramos-Alonso L, Romero AM, Martinez-Pastor MT, Jiang S-C, Sah-Teli SK, Li L, Bentrem D, Lopaschuk G, Ben-Sahra I, O’Halloran TV, Shilatifard A, Puig S, Bergelson J, Koivunen P, Ardehali H. 2023. Iron drives anabolic metabolism through active histone demethylation and mTORC1. Nature Cell Biology 25:1478–1494. 37749225 10.1038/s41556-023-01225-6 PMC11407783

[71] Lane A, Gokhale A, Werner E, Roberts A, Freeman A, Roberts B, Faundez V. 2022. Sulfur- and phosphorus-standardized metal quantification of biological specimens using inductively coupled plasma mass spectrometry. STAR Protoc 3:101334. 35496782 10.1016/j.xpro.2022.101334 PMC9047006

[72] Schaechter M, MaalØe O, Kjeldgaard NO. 1958. Dependency on medium and temperature of cell size and chemical composition during balanced growth of *Salmonella typhimurium*. Microbiology 19:592–606. 10.1099/00221287-19-3-592 13611202

[73] Kjeldgaard NO, MaalØe O, Schaechter M. 1958. The transition between different physiological states during balanced growth of *Salmonella typhimurium*. Microbiology 19:607–616. 10.1099/00221287-19-3-607 13611203

[74] Mulyukin AL, Sorokin VV, Loiko NG, Suzina NE, Duda VI, Vorob’eva EA, El’-Registan GI. 2002. Comparative study of the elemental composition of vegetative and resting microbial cells. Microbiology 71:31–40. 11910805

[75] Sivakumar P, Fernández-Bravo A, Taleh L, Biddle JF, Melikechi N. 2015. Detection and classification of live and dead *Escherichia coli* by laser-induced breakdown spectroscopy. Astrobiology 15:144–153. 25683088 10.1089/ast.2014.1181 PMC4323123

[76] Sartorio Mariana G, Valguarnera E, Hsu F-F, Feldman Mario F. 2022. Lipidomics analysis of outer membrane vesicles and elucidation of the inositol phosphoceramide biosynthetic pathway in *Bacteroides thetaiotaomicron*. Microbiology Spectrum 10:e00634–21. 10.1128/spectrum.00634-21 PMC8791184 35080445

[77] Ryan E, Gonzalez Pastor B, Gethings LA, Clarke DJ, Joyce SA. 2023. Lipidomic analysis reveals differences in *Bacteroides* species driven largely by plasmalogens, glycerophosphoinositols and certain sphingolipids. Metabolites 13:360. 36984802 10.3390/metabo13030360 PMC10056535

[78] Rizza V, Tucker Anne N, White David C. 1970. Lipids of *Bacteroides melaninogenicus*. Journal of Bacteriology 101:84–91. 5411759 10.1128/jb.101.1.84-91.1970 PMC250454

[79] Withers H, Swift S, Williams P. 2001. Quorum sensing as an integral component of gene regulatory networks in Gram-negative bacteria. Current Opinion in Microbiology 4:186–193. 11282475 10.1016/s1369-5274(00)00187-9

[80] Otaru N, Ye K, Mujezinovic D, Berchtold L, Constancias F, Cornejo FA, Krzystek A, de Wouters T, Braegger C, Lacroix C, Pugin B. 2021. GABA production by human intestinal Bacteroides spp.: Prevalence, regulation, and role in acid stress tolerance. Frontiers in Microbiology 12. 10.3389/fmicb.2021.656895 PMC8082179 33936013

[81] Cohen LJ, Kang HS, Chu J, Huang YH, Gordon EA, Reddy BV, Ternei MA, Craig JW, Brady SF. 2015. Functional metagenomic discovery of bacterial effectors in the human microbiome and isolation of commendamide, a GPCR G2A/132 agonist. Proc Natl Acad Sci U S A 112:E4825–34. 26283367 10.1073/pnas.1508737112 PMC4568208

[82] Koprivnjak T, Zhang D, Ernst CM, Peschel A, Nauseef WM, Weiss JP. 2011. Characterization of *Staphylococcus aureus* cardiolipin synthases 1 and 2 and their contribution to accumulation of cardiolipin in stationary phase and within phagocytes. Journal of Bacteriology 193:4134–4142. 21665977 10.1128/JB.00288-11 PMC3147675

[83] Mirretta Barone C, Heaver SL, Gruber L, Zundel F, Vu DL, Ley RE. 2024. Spatially resolved lipidomics shows conditional transfer of lipids produced by <em>Bacteroides</em> thetaiotaomicron into the mouse gut. Cell Host & Microbe 32:1025–1036.e5. 38795710 10.1016/j.chom.2024.04.021

[84] An D, Na C, Bielawski J, Hannun YA, Kasper DL. 2011. Membrane sphingolipids as essential molecular signals for Bacteroides survival in the intestine. Proceedings of the National Academy of Sciences 108:4666–4671. 10.1073/pnas.1001501107 PMC3063596 20855611

[85] Frankfater CF, Sartorio MG, Valguarnera E, Feldman MF, Hsu F-F. 2023. Lipidome of the Bacteroides Genus Containing New Peptidolipid and Sphingolipid Families Revealed by Multiple-Stage Mass Spectrometry. Biochemistry 62:1160–1180. 36880942 10.1021/acs.biochem.2c00664 PMC12085236

[86] Hines KM, Xu L. 2019. Lipidomic consequences of phospholipid synthesis defects in *Escherichia coli* revealed by HILIC-ion mobility-mass spectrometry. Chemistry and Physics of Lipids 219:15–22. 30660747 10.1016/j.chemphyslip.2019.01.007 PMC6438183

[87] Czolkoss S, Fritz C, Hölzl G, Aktas M. 2016. Two distinct cardiolipin synthases operate in *Agrobacterium tumefaciens*. PLOS ONE 11:e0160373. 27472399 10.1371/journal.pone.0160373 PMC4966929

[88] Tsai M, Ohniwa RL, Kato Y, Takeshita SL, Ohta T, Saito S, Hayashi H, Morikawa K. 2011. *Staphylococcus aureus* requires cardiolipin for survival under conditions of high salinity. BMC Microbiology 11:13. 21241511 10.1186/1471-2180-11-13 PMC3030509

[89] Liu J, Ryabichko S, Bogdanov M, Fackelmayer OJ, Dowhan W, Krulwich TA. 2014. Cardiolipin is dispensable for oxidative phosphorylation and non-fermentative growth of alkaliphilic Bacillus pseudofirmus OF4. J Biol Chem 289:2960–71. 24338478 10.1074/jbc.M113.536193 PMC3908427

[90] Kusaka J, Shuto S, Imai Y, Ishikawa K, Saito T, Natori K, Matsuoka S, Hara H, Matsumoto K. 2016. Septal localization by membrane targeting sequences and a conserved sequence essential for activity at the COOH-terminus of *Bacillus subtilis* cardiolipin synthase. Research in Microbiology 167:202–214. 26708983 10.1016/j.resmic.2015.11.004

[91] Shibuya I, Yamagoe S, Miyazaki C, Matsuzaki H, Ohta A. 1985. Biosynthesis of novel acidic phospholipid analogs in *Escherichia coli*. Journal of Bacteriology 161:473–477. 3918012 10.1128/jb.161.2.473-477.1985 PMC214906

[92] Walderhaug MO, Dosch DC, Epstein W. 1987. Potassium Transport in Bacteria, p 85–130. In Rosen BP, Silver S (ed), Ion Transport in Prokaryotes doi:10.1016/B978-0-12-596935-2.50005-0. Academic Press.

[93] Whatmore AM, Chudek JA, Reed RH. 1990. The effects of osmotic upshock on the intracellular solute pools of *Bacillus subtilis*. Microbiology 136:2527–2535. 10.1099/00221287-136-12-2527 2127802

[94] Epstein W. 1986. Osmoregulation by potassium transport in *Escherichia coli*. FEMS Microbiology Reviews 2:73–78.

[95] Christian JHB, Waltho JA. 1964. The composition of *Staphylococcus aureus* in relation to the water activity of the growth medium. Microbiology 35:205–213. 10.1099/00221287-35-2-205 14179669

[96] Jagannathan S. 1994. Characterization of proton and water permeability across model membrane vesicles and proteoliposomes. 9600672. Clemson University, South Carolina, USA.

[97] Kato S, Tobe H, Matsubara H, Sawada M, Sasaki Y, Fukiya S, Morita N, Yokota A. 2019. The membrane phospholipid cardiolipin plays a pivotal role in bile acid adaptation by Lactobacillus gasseri JCM1131T. Biochimica et Biophysica Acta (BBA) - Molecular and Cell Biology of Lipids 1864:403–412. 29883797 10.1016/j.bbalip.2018.06.004

[98] Dimroth P. 1994. Bacterial sodium ion-coupled energetics. Antonie Van Leeuwenhoek 65:381–95. 7832594 10.1007/BF00872221

[99] Mulkidjanian AY, Dibrov P, Galperin MY. 2008. The past and present of sodium energetics: may the sodium-motive force be with you. Biochim Biophys Acta 1777:985–92. 18485887 10.1016/j.bbabio.2008.04.028 PMC2695506

[100] Ren Q, Paulsen IT. 2009. Transport, Solute, p 529–544. *In* Schaechter M (ed), Encyclopedia of Microbiology (Third Edition) doi:10.1016/B978-012373944-5.00107-3. Academic Press, Oxford.

[101] van de Vossenberg JL, Ubbink-Kok T, Elferink MG, Driessen AJ, Konings WN. 1995. Ion permeability of the cytoplasmic membrane limits the maximum growth temperature of bacteria and archaea. Mol Microbiol 18:925–32. 8825096 10.1111/j.1365-2958.1995.18050925.x

[102] Deamer DW, Bramhall J. 1986. Permeability of lipid bilayers to water and ionic solutes. Chemistry and Physics of Lipids 40:167–188. 2427233 10.1016/0009-3084(86)90069-1

[103] Hopfer U, Lehninger AL, Lennarz WJ. 1970. The effect of the polar moiety of lipids on the ion permeability of bilayer membranes. The Journal of Membrane Biology 2:41–58. 24174136 10.1007/BF01869849

[104] Russell JB. 1987. A proposed mechanism of monensin action in inhibiting ruminant bacterial growth: Effects on ion flux and proton motive force. Journal of Animal Science 64:1519–1525. 3583956 10.2527/jas1987.6451519x

[105] Guffanti AA, Davidson LF, Mann TM, Krulwich TA. 1979. Nigericin-induced death of an acidophilic bacterium. Microbiology 114:201–206. 10.1099/00221287-114-1-201 42667

[106] Ouellette SP, Fisher-Marvin LA, Harpring M, Lee J, Rucks EA, Cox JV. 2022. Localized cardiolipin synthesis is required for the assembly of MreB during the polarized cell division of *Chlamydia trachomatis*. PLOS Pathogens 18:e1010836. 36095021 10.1371/journal.ppat.1010836 PMC9499288

[107] Kurita K, Kato F, Shiomi D. 2020. Alteration of membrane fluidity or phospholipid composition perturbs rotation of MreB complexes in Escherichia coli. Frontiers in Molecular Biosciences 7. 10.3389/fmolb.2020.582660 PMC7719821 33330621

[108] Dong H, Zhang Z, Tang X, Huang S, Li H, Peng B, Dong C. 2016. Structural insights into cardiolipin transfer from the Inner membrane to the outer membrane by PbgA in Gram-negative bacteria. Scientific Reports 6:30815–30815. 27487745 10.1038/srep30815 PMC4973235

[109] Bogdanov M. 2023. The power and challenge of lipid (a)symmetry across the membrane and cell. Emerging Topics in Life Sciences 7:1–6. 36988943 10.1042/ETLS20220088 PMC10725184

[110] Bogdanov M. 2023. Renovating a double fence with or without notifying the next door and across the street neighbors: why the biogenic cytoplasmic membrane of Gram-negative bacteria display asymmetry?. Emerging Topics in Life Sciences 7:137–150. 36960750 10.1042/ETLS20230042 PMC10725183

[111] Lande MB, Donovan JM, Zeidel ML. 1995. The relationship between membrane fluidity and permeabilities to water, solutes, ammonia, and protons. Journal of General Physiology 106:67–84. 7494139 10.1085/jgp.106.1.67 PMC2229255

[112] Neidleman SL. 1987. Effects of temperature on lipid unsaturation. Biotechnology and Genetic Engineering Reviews 5:245–268. 3314900 10.1080/02648725.1987.10647839

[113] Yano Y, Nakayama A, Ishihara K, Saito H. 1998. Adaptive changes in membrane lipids of barophilic bacteria in response to changes in growth pressure. Applied and Environmental Microbiology 64:479–485. 16349499 10.1128/aem.64.2.479-485.1998 PMC106069

[114] Huffer S, Clark ME, Ning JC, Blanch HW, Clark DS. 2011. Role of alcohols in growth, lipid composition, and membrane fluidity of yeasts, bacteria, and archaea. Applied and Environmental Microbiology 77:6400–6408. 21784917 10.1128/AEM.00694-11 PMC3187150

[115] Keweloh H, Heipieper HJ. 1996. Trans unsaturated fatty acids in bacteria. Lipids 31:129–137. 8835399 10.1007/BF02522611

[116] Altabe SG, Aguilar P, Caballero GM, de Mendoza D. 2003. The *Bacillus subtilis* acyl lipid desaturase is a delta5 desaturase. J Bacteriol 185:3228–31. 12730185 10.1128/JB.185.10.3228-3231.2003 PMC154086

[117] Schweizer HP, Choi K-H. 2011. *Pseudomonas aeruginosa* aerobic fatty acid desaturase DesB is important for virulence factor production. Archives of Microbiology 193:227–234. 21184216 10.1007/s00203-010-0665-6

[118] García-Bayona L, Comstock LE. 2019. Streamlined genetic manipulation of diverse *Bacteroides* and *Parabacteroides* isolates from the human gut microbiota. mBio 10. 10.1128/mBio.01762-19 PMC6692515 31409684

[119] Rajeev L, Segall A, Gardner J. 2007. The *Bacteroides* NBU1 integrase performs a homology-independent strand exchange to form a holliday junction intermediate. J Biol Chem 282:31228–37. 17766246 10.1074/jbc.M705370200

[120] Hallgren J, Tsirigos KD, Pedersen MD, Almagro Armenteros JJ, Marcatili P, Nielsen H, Krogh A, Winther O. 2022. DeepTMHMM predicts alpha and beta transmembrane proteins using deep neural networks. bioRxiv doi:10.1101/2022.04.08.487609:2022.04.08.487609.

[121] Bäckhed F, Ley RE, Sonnenburg JL, Peterson DA, Gordon JI. 2005. Host-bacterial mutualism in the human intestine. Science 307:1915–1920. 15790844 10.1126/science.1104816

[122] Adak A, Khan MR. 2019. An insight into gut microbiota and its functionalities. Cellular and Molecular Life Sciences 76:473–493. 30317530 10.1007/s00018-018-2943-4 PMC11105460

[123] Lu S, Wang J, Chitsaz F, Derbyshire MK, Geer RC, Gonzales NR, Gwadz M, Hurwitz DI, Marchler GH, Song JS, Thanki N, Yamashita RA, Yang M, Zhang D, Zheng C, Lanczycki CJ, Marchler-Bauer A. 2020. CDD/SPARCLE: The conserved domain database in 2020. Nucleic Acids Res 48:D265–d268. 31777944 10.1093/nar/gkz991 PMC6943070

[124] Saha CK, Sanches Pires R, Brolin H, Delannoy M, Atkinson GC. 2020. FlaGs and webFlaGs: Discovering novel biology through the analysis of gene neighbourhood conservation. Bioinformatics 37:1312–1314. 10.1093/bioinformatics/btaa788 PMC8189683 32956448

[125] Wang J, Barger K. 2024. ipolygrowth: An R package to calculate individual growth curve parameters from bacterial time series data. Microbiology Resource Announcements 13:e00662–24. 39329416 10.1128/mra.00662-24 PMC11556007

[126] McLaughlin M, Fiebig A, Crosson S. 2023. XRE transcription factors conserved in Caulobacter and φCbK modulate adhesin development and phage production. PLOS Genetics 19:e1011048. 37972151 10.1371/journal.pgen.1011048 PMC10688885

[127] Ducret A, Quardokus EM, Brun YV. 2016. MicrobeJ, a tool for high throughput bacterial cell detection and quantitative analysis. Nature Microbiology 1:16077. 10.1038/nmicrobiol.2016.77 PMC5010025 27572972

[128] Sakol N, Egawa A, Fujiwara T. 2020. Gadolinium complexes as contrast agent for cellular NMR spectroscopy. Int J Mol Sci 21:4042. 32516957 10.3390/ijms21114042 PMC7312942

[129] Aime S, Cabella C, Colombatto S, Geninatti Crich S, Gianolio E, Maggioni F. 2002. Insights into the use of paramagnetic Gd(III) complexes in MR-molecular imaging investigations. Journal of Magnetic Resonance Imaging 16:394–406. 12353255 10.1002/jmri.10180

[130] Basso JTR, Jones KA, Jacobs KR, Christopher CJ, Fielland HB, Campagna SR, Buchan A. 2022. Growth Substrate and Prophage Induction Collectively Influence Metabolite and Lipid Profiles in a Marine Bacterium. mSystems 7:e0058522. 35972149 10.1128/msystems.00585-22 PMC9600351

[131] Woodall B, Fozo EM, Campagna SR. 2023. Dual stable isotopes enhance lipidomic studies in bacterial model organism Enterococcus faecalis. Anal Bioanal Chem 415:3593–3605. 37204445 10.1007/s00216-023-04750-3

[132] Tsugawa H, Ikeda K, Takahashi M, Satoh A, Mori Y, Uchino H, Okahashi N, Yamada Y, Tada I, Bonini P, Higashi Y, Okazaki Y, Zhou Z, Zhu Z-J, Koelmel J, Cajka T, Fiehn O, Saito K, Arita M, Arita M. 2020. A lipidome atlas in MS-DIAL 4. Nature Biotechnology 38:1159–1163. 10.1038/s41587-020-0531-2 32541957

[133] Tsugawa H, Nakabayashi R, Mori T, Yamada Y, Takahashi M, Rai A, Sugiyama R, Yamamoto H, Nakaya T, Yamazaki M, Kooke R, Bac-Molenaar JA, Oztolan-Erol N, Keurentjes JJB, Arita M, Saito K. 2019. A cheminformatics approach to characterize metabolomes in stable-isotope-labeled organisms. Nature Methods 16:295–298. 30923379 10.1038/s41592-019-0358-2

[134] Lai Z, Tsugawa H, Wohlgemuth G, Mehta S, Mueller M, Zheng Y, Ogiwara A, Meissen J, Showalter M, Takeuchi K, Kind T, Beal P, Arita M, Fiehn O. 2018. Identifying metabolites by integrating metabolome databases with mass spectrometry cheminformatics. Nature Methods 15:53–56. 29176591 10.1038/nmeth.4512 PMC6358022

[135] Tsugawa H, Cajka T, Kind T, Ma Y, Higgins B, Ikeda K, Kanazawa M, VanderGheynst J, Fiehn O, Arita M. 2015. MS-DIAL: data-independent MS/MS deconvolution for comprehensive metabolome analysis. Nat Methods 12:523–6. 25938372 10.1038/nmeth.3393 PMC4449330

[136] Friedman JH, Hastie T, Tibshirani R. 2010. Regularization paths for generalized linear models via coordinate descent. Journal of Statistical Software 33:1 – 22. 20808728 PMC2929880

[137] Tay JK, Narasimhan B, Hastie T. 2023. Elastic net regularization paths for all generalized linear models. Journal of Statistical Software 106:1 – 31. 37138589 10.18637/jss.v106.i01 PMC10153598

[138] Mason Allison R, Johnson JG, Krampen J, Nguyen Jennifer NT, Balunas Marcy J, Schloss Patrick D. 2025. mpactR: An R adaptation of the metabolomics peak analysis computational tool (MPACT) for use in reproducible data analysis pipelines. Microbiology Resource Announcements 14:e00997–24. 39812609 10.1128/mra.00997-24 PMC11812337

[139] Wang M, Carver JJ, Phelan VV, Sanchez LM, Garg N, Peng Y, Nguyen DD, Watrous J, Kapono CA, Luzzatto-Knaan T, Porto C, Bouslimani A, Melnik AV, Meehan MJ, Liu W-T, Crüsemann M, Boudreau PD, Esquenazi E, Sandoval-Calderón M, Kersten RD, Pace LA, Quinn RA, Duncan KR, Hsu C-C, Floros DJ, Gavilan RG, Kleigrewe K, Northen T, Dutton RJ, Parrot D, Carlson EE, Aigle B, Michelsen CF, Jelsbak L, Sohlenkamp C, Pevzner P, Edlund A, McLean J, Piel J, Murphy BT, Gerwick L, Liaw C-C, Yang Y-L, Humpf H-U, Maansson M, Keyzers RA, Sims AC, Johnson AR, Sidebottom AM, Sedio BE, 2016. Sharing and community curation of mass spectrometry data with Global Natural Products Social Molecular Networking. Nature Biotechnology 34:828–837. 10.1038/nbt.3597 PMC5321674 27504778

[140] Shannon P, Markiel A, Ozier O, Baliga NS, Wang JT, Ramage D, Amin N, Schwikowski B, Ideker T. 2003. Cytoscape: A software environment for integrated models of biomolecular interaction networks. Genome Res 13:2498–504. 14597658 10.1101/gr.1239303 PMC403769

[141] Pang Z, Lu Y, Zhou G, Hui F, Xu L, Viau C, Spigelman Aliya F, MacDonald Patrick E, Wishart David S, Li S, Xia J. 2024. MetaboAnalyst 6.0: Towards a unified platform for metabolomics data processing, analysis and interpretation. Nucleic Acids Research 52:W398–W406. 38587201 10.1093/nar/gkae253 PMC11223798

[142] Team RDC. 2013. R: A language and environment for statistical computing, R Foundation for Statistical Computing, http://www.R-project.org/.

[143] Wickham H. 2016. ggplot2: Elegant graphics for data analysis, Springer-Verlag, New York. https://ggplot2.tidyverse.org.

[144] Wong B. 2011. Points of view: Color blindness. Nature Methods 8:441–441. 37452153 10.1038/s41592-023-01974-0

[145] Ou J. 2021. colorBlindness: Safe color set for color blindness, https://CRAN.R-project.org/package=colorBlindnes.

[146] Neuwirth E. 2022. RColorBrewer: ColorBrewer palettes, https://CRAN.R-project.org/package=RColorBrewer.

[147] Vu VQ, Friendly M. 2024. ggbiplot: A grammar of graphics implementation of biplots, https://CRAN.R-project.org/package=ggbiplot.

[148] Pedersen TL. 2024. ggforce: Accelerating ‘ggplot2’, https://CRAN.R-project.org/package=ggforce.

[149] Gu Z, Gu L, Eils R, Schlesner M, Brors B. 2014. circlize implements and enhances circular visualization in R. Bioinformatics 30:2811–2. 24930139 10.1093/bioinformatics/btu393

[150] Gu Z, Eils R, Schlesner M. 2016. Complex heatmaps reveal patterns and correlations in multidimensional genomic data. Bioinformatics 32:2847–9. 27207943 10.1093/bioinformatics/btw313

[151] Neitmann T. 2020. mdthemes: Markdown themes for ‘ggplot2’, https://CRAN.R-project.org/package=mdthemes.

[152] C W B W. 2022. ggtext: Improved text rendering support for ‘ggplot2’, https://CRAN.R-project.org/package=ggtext.

[153] Wickham H, Bryan J. 2023. readxl: Read excel files, https://CRAN.R-project.org/package=readxl.

[154] Wickham H, Vaughan D, Girlich M. 2024. tidyr: Tidy messy data, https://CRAN.R-project.org/package=tidyr.

[155] Bengtsson H. 2024. matrixStats: Functions that apply to rows and columns of matrices (and to vectors), https://CRAN.R-project.org/package=matrixStats.

[156] Barrett T, Dowle M, Srinivasan A, Gorecki J, Chirico M, Hocking T. 2024. data.table: Extension of `data.frame`, https://CRAN.R-project.org/package=data.table.

[157] Bache SM, Wickham H. 2022. magrittr: A forward-pipe operator for R, https://cran.r-project.org/web/packages/magrittr/vignettes/magrittr.html.

[158] Wickham H, Pedersen TL, Seidel D. 2023. scales: Scale functions for visualization, https://CRAN.R-project.org/package=scales.

[159] Wang L-G, Lam TT-Y, Xu S, Dai Z, Zhou L, Feng T, Guo P, Dunn CW, Jones BR, Bradley T, Zhu H, Guan Y, Jiang Y, Yu G. 2019. Treeio: An R package for phylogenetic tree input and output with richly annotated and associated data. Molecular Biology and Evolution 37:599–603. 10.1093/molbev/msz240 PMC6993851 31633786

[160] Yu G, Smith DK, Zhu H, Guan Y, Lam TT-Y. 2017. ggtree: An R package for visualization and annotation of phylogenetic trees with their covariates and other associated data. Methods in Ecology and Evolution 8:28–36.

[161] Xu S, Li L, Luo X, Chen M, Tang W, Zhan L, Dai Z, Lam TT, Guan Y, Yu G. 2022. Ggtree: A serialized data object for visualization of a phylogenetic tree and annotation data. iMeta 1:e56. 38867905 10.1002/imt2.56 PMC10989815

[162] Bates D, Mächler M, Bolker B, Walker S. 2015. Fitting linear mixed-effects models using lme4. J of Statistical Software 67:1–48.

[163] Kuznetsova A, Brockhoff PB, Christensen RHB. 2017. lmerTest package: Tests in linear mixed effects models. J of Statistical Software 82:1–26.

[164] A K. 2023. ggpubr: ‘ggplot2’ based publication ready plots, https://CRAN.R-project.org/package=ggpubr.

[165] Yurekten O, Payne T, Tejera N, Amaladoss FX, Martin C, Williams M, O’Donovan C. 2023. MetaboLights: open data repository for metabolomics. Nucleic Acids Research 52:D640–D646. 10.1093/nar/gkad1045 PMC10767962 37971328

[166] Vingadassalom D, Kolb A, Mayer C, Rybkine T, Collatz E, Podglajen I. 2005. An unusual primary sigma factor in the Bacteroidetes phylum. Mol Microbiol 56:888–902. 15853878 10.1111/j.1365-2958.2005.04590.x

[167] Loferer-Krössbacher M, Klima J, Psenner R. 1998. Determination of bacterial cell dry mass by transmission electron microscopy and densitometric image analysis. Appl Environ Microbiol 64:688–94. 9464409 10.1128/aem.64.2.688-694.1998 PMC106103

[168] Epanechnikov VA. 1969. Non-parametric estimation of a multivariate probability density. Theory of Probability & Its Applications 14:153–158.

[169] Poynton EF, van Santen JA, Pin M, Contreras MM, McMann E, Parra J, Showalter B, Zaroubi L, Duncan Katherine R, Linington RG. 2024. The Natural Products Atlas 3.0: Extending the database of microbially derived natural products. Nucleic Acids Research 53:D691–D699. 10.1093/nar/gkae1093 PMC11701703 39588755

[170] Wang F, Allen D, Tian S, Oler E, Gautam V, Greiner R, Metz Thomas O, Wishart David S. 2022. CFM-ID 4.0 – a web server for accurate MS-based metabolite identification. Nucleic Acids Research 50:W165–W174. 35610037 10.1093/nar/gkac383 PMC9252813

[171] Conroy MJ, Andrews RM, Andrews S, Cockayne L, Dennis Edward A, Fahy E, Gaud C, Griffiths William J, Jukes G, Kolchin M, Mendivelso K, Lopez-Clavijo Andrea F, Ready C, Subramaniam S, O’Donnell Valerie B. 2023. LIPID MAPS: update to databases and tools for the lipidomics community. Nucleic Acids Research 52:D1677–D1682. 10.1093/nar/gkad896 PMC10767878 37855672

