# Supplementary material for: Non-redundant cardiolipin synthases shape membrane composition and support stress resilience in Bacteroides fragilis
Source: bioRxiv. 2025 May 19:2025.05.12.653583. Preprint. [Version 3] doi: 10.1101/2025.05.12.653583 (PMC12132519; doi:10.1101/2025.05.12.653583)
Supplement: Supplement 9 [file NIHPP2025.05.12.653583v3-supplement-9.pdf]

bioRxiv preprint doi: https://doi.org/10.1101/2025.05.12.653583; this version posted May 19, 2025. The copyright holder for this preprint (which was not certified by peer review) is the author/funder, who has granted bioRxiv a license to display the preprint in perpetuity. It is made available under aCC-BY 4.0 International license.

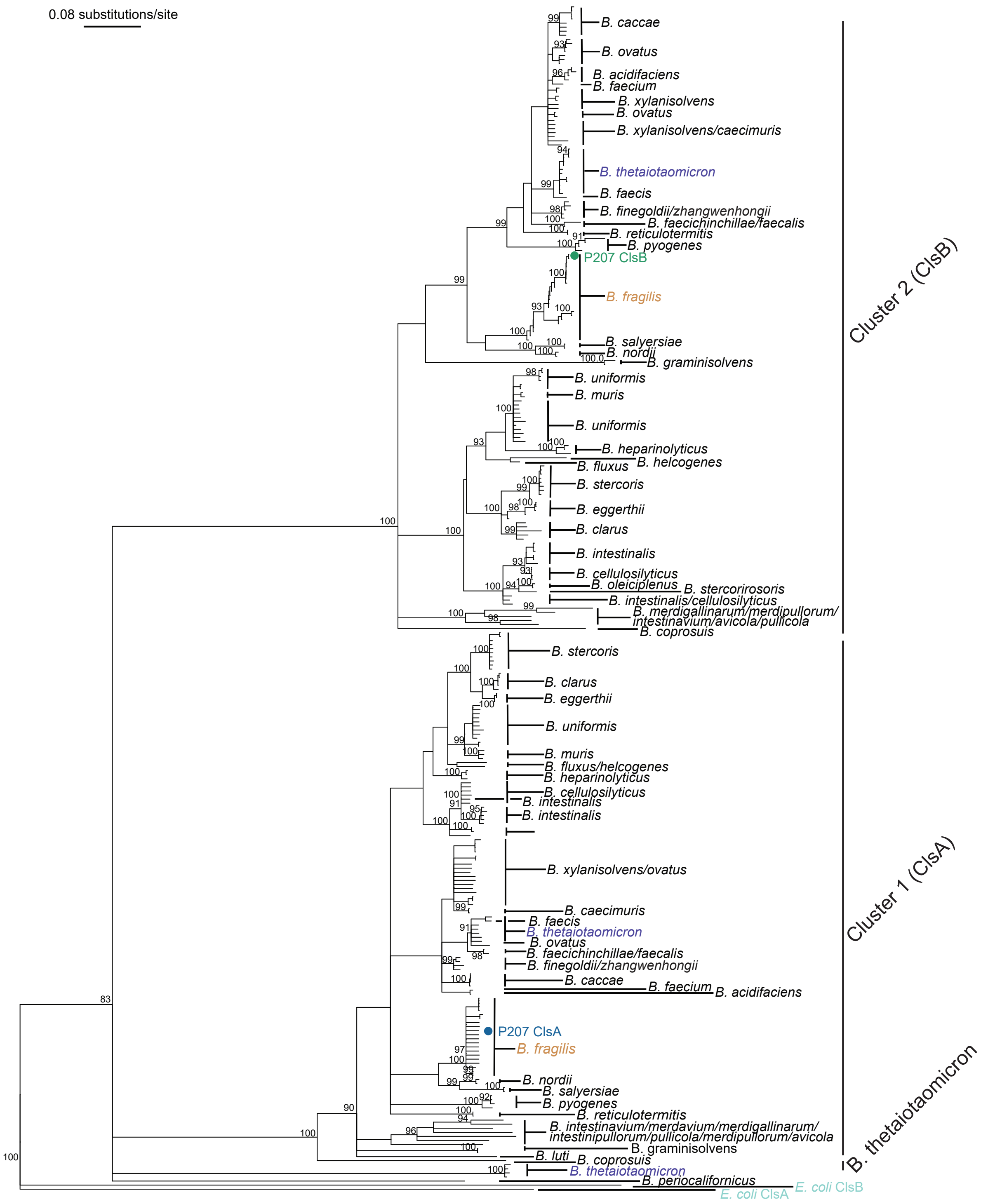

Figure S1. Neighbor-joining tree of cardiolipin synthases from *Bacteroides* species. Clusters 1 (ClsA), 2 (ClsB) and 3 (not named) of *Bacteroides* Cls proteins are indicated. *E. coli* ClsA was designated as an outgroup. ClsA and ClsB of *B. fragilis* P207 are colored in blue and green, respectively. Other strains

bioRxiv preprint doi: https://doi.org/10.1101/2025.05.12.653583; this version posted May 19, 2025. The copyright holder for this preprint (which was not certified by peer review) is the author/funder, who has granted bioRxiv a license to display the preprint in perpetuity. It is made available under aCC-BY 4.0 International license.

of *B. fragilis, B. thetaiotaomicron*, and *E. coli* are colored in gold, purple and light blue, respectively. Taxa labels with similar tree positions are combined for clarity at times (i.e., *B. xylanisolvens/caecimuris*). Key indicates the rate of amino acid substitutions per site. Bootstrap values are generally shown only if ≥90.

bioRxiv preprint doi: https://doi.org/10.1101/2025.05.12.653583; this version posted May 19, 2025. The copyright holder for this preprint (which was not certified by peer review) is the author/funder, who has granted bioRxiv a license to display the preprint in perpetuity. It is made available under aCC-BY 4.0 International license.

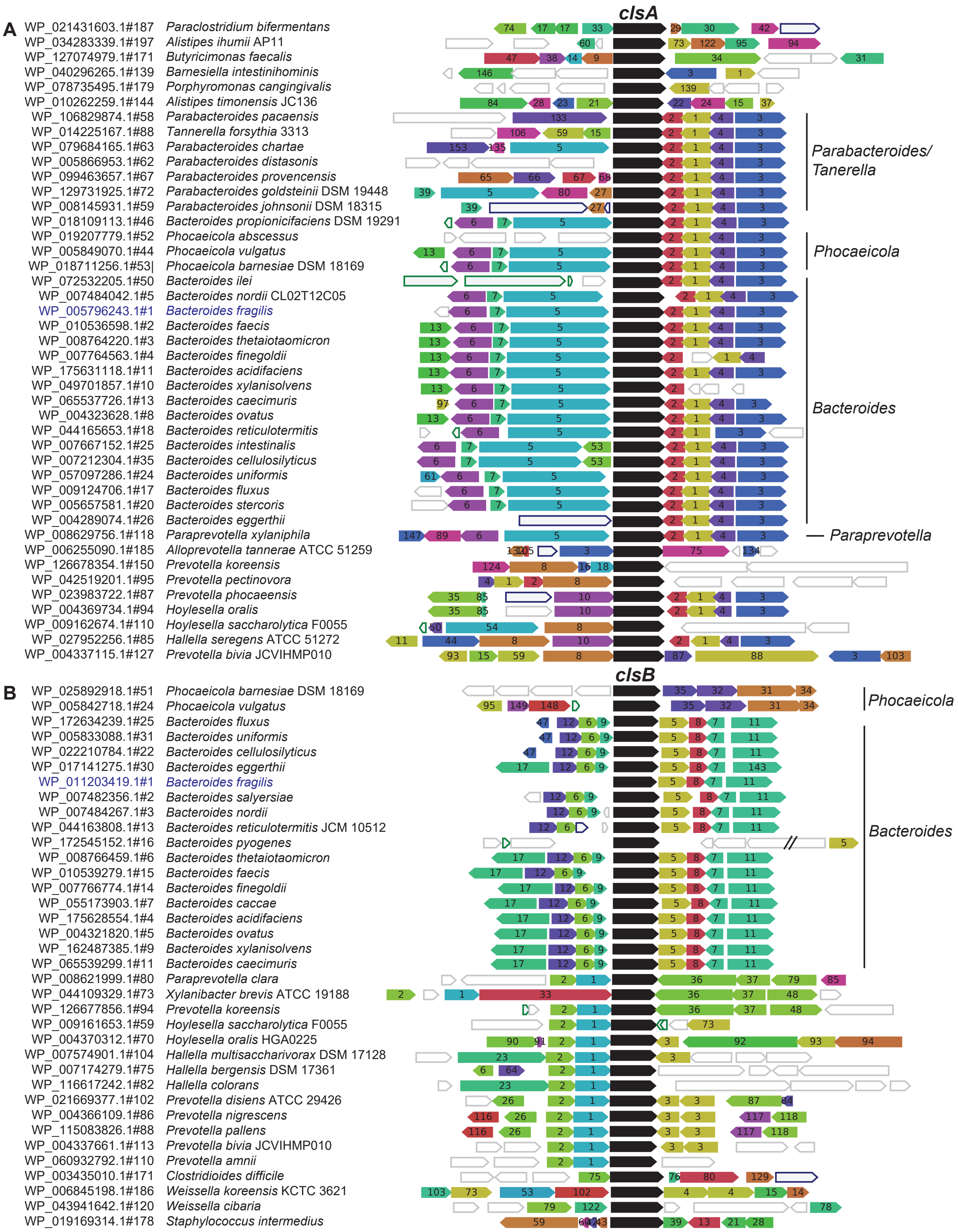

bioRxiv preprint doi: https://doi.org/10.1101/2025.05.12.653583; this version posted May 19, 2025. The copyright holder for this preprint (which was not certified by peer review) is the author/funder, who has granted bioRxiv a license to display the preprint in perpetuity. It is made available under aCC-BY 4.0 International license.

Figure S2. Genomic neighborhood synteny indicates the ancestral introduction of *clsA* and *clsB* into the *Bacteroides.* Curated WebFlags2 output of (**A.**) *clsA* and (**B.**) *clsB* genomic neighborhoods. *B. fragilis* is colored blue. Genera of interest with similar levels of gene conservation are indicated at right.

bioRxiv preprint doi: https://doi.org/10.1101/2025.05.12.653583; this version posted May 19, 2025. The copyright holder for this preprint (which was not certified by peer review) is the author/funder, who has granted bioRxiv a license to display the preprint in perpetuity. It is made available under aCC-BY 4.0 International license.

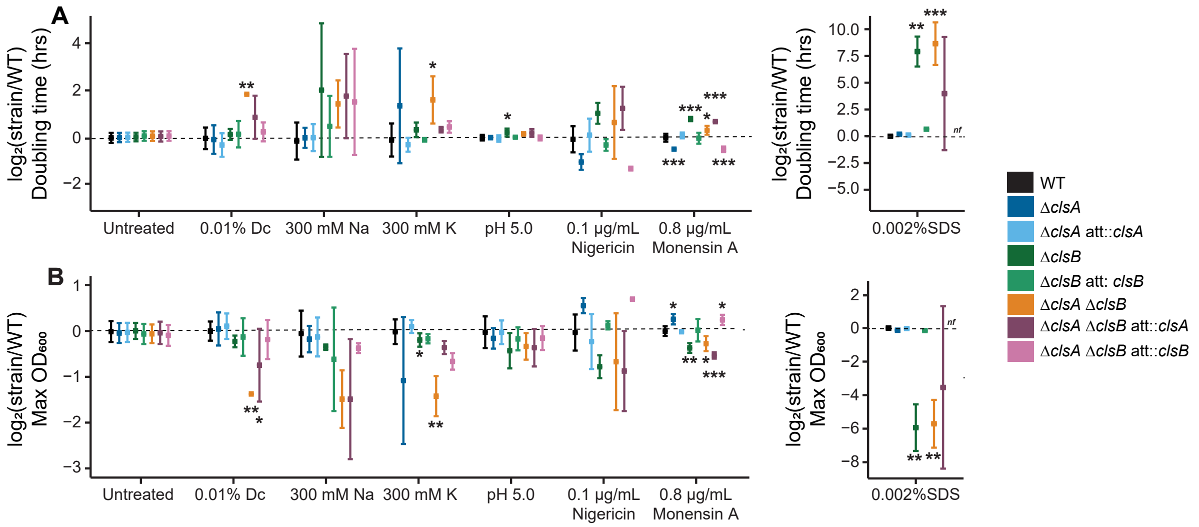

Figure S3. Loss of cardiolipin synthase activity results in growth defects under stress. A., B. $\log_2$(fold change) of (**A.**) doubling time and (**B.**) maximum OD_{600} comparing *B. fragilis* wild-type (WT) and *cls* mutants to the average of WT in that condition. Metrics were calculated from 24-h growth curves in BHIS medium alone or containing 0.01% deoxycholate (Dc), 0.002% sodium dodecyl sulfate (SDS), 300 mM sodium (Na^+), 300 mM potassium (K^+), and 0.01 µg/mL nigericin and 0.8 µg/mL monensin A. Any doubling time ≥100 h was considered as no growth. The dashed horizontal line indicates no change from the average of WT. Strain are colored with blue (*clsA-related*), green (*clsB*-related) and orange/purple (*clsA clsB*-related). Black indicates WT. The vertical lines indicate mean; the ends of the whiskers ± standard deviation (linear regression compared to WT). Three biological replicates for each condition were run, each run in technical triplicate. nf, no accurate fit of growth data by the polynomial was found. Unadjusted p-values are shown; *, $p < 0.05$; **, $p < 0.01$; ***, $p < 0.001$.

bioRxiv preprint doi: https://doi.org/10.1101/2025.05.12.653583; this version posted May 19, 2025. The copyright holder for this preprint (which was not certified by peer review) is the author/funder, who has granted bioRxiv a license to display the preprint in perpetuity. It is made available under aCC-BY 4.0 International license.

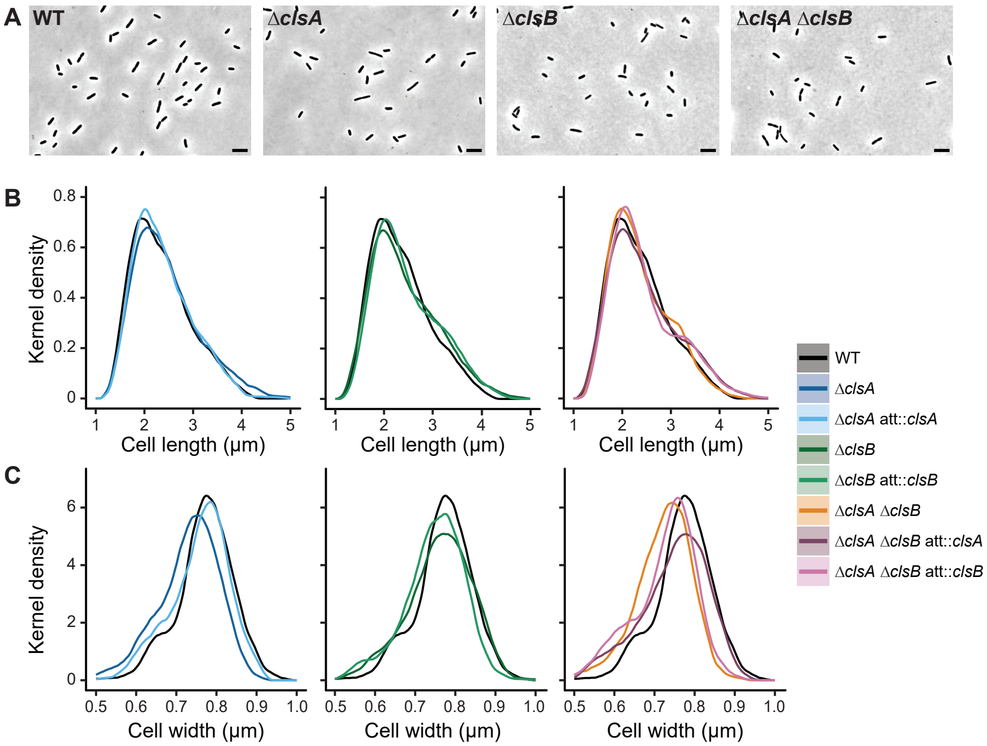

1308

1309 **Figure S4. Loss of cardiolipin synthase activity is associated with defects in cell width. A.** Phase-

1310 contrast microscopy images of *B. fragilis* P207 WT, Δ*clsA*, Δ*clsB* and Δ*clsA* Δ*clsB* strains. Scale bars, 5 µm.

1311 **B., C.** Distributions estimated by kernel density of (**B.**) cell length and (**C.**) cell width. Results are plotted by

1312 strain with blue, green and orange/purple indicating *clsA*-, *clsB*- and *clsA clsB*-related strains, respectively.

1313 Black indicates WT.

1314

bioRxiv preprint doi: https://doi.org/10.1101/2025.05.12.653583; this version posted May 19, 2025. The copyright holder for this preprint (which was not certified by peer review) is the author/funder, who has granted bioRxiv a license to display the preprint in perpetuity. It is made available under aCC-BY 4.0 International license.

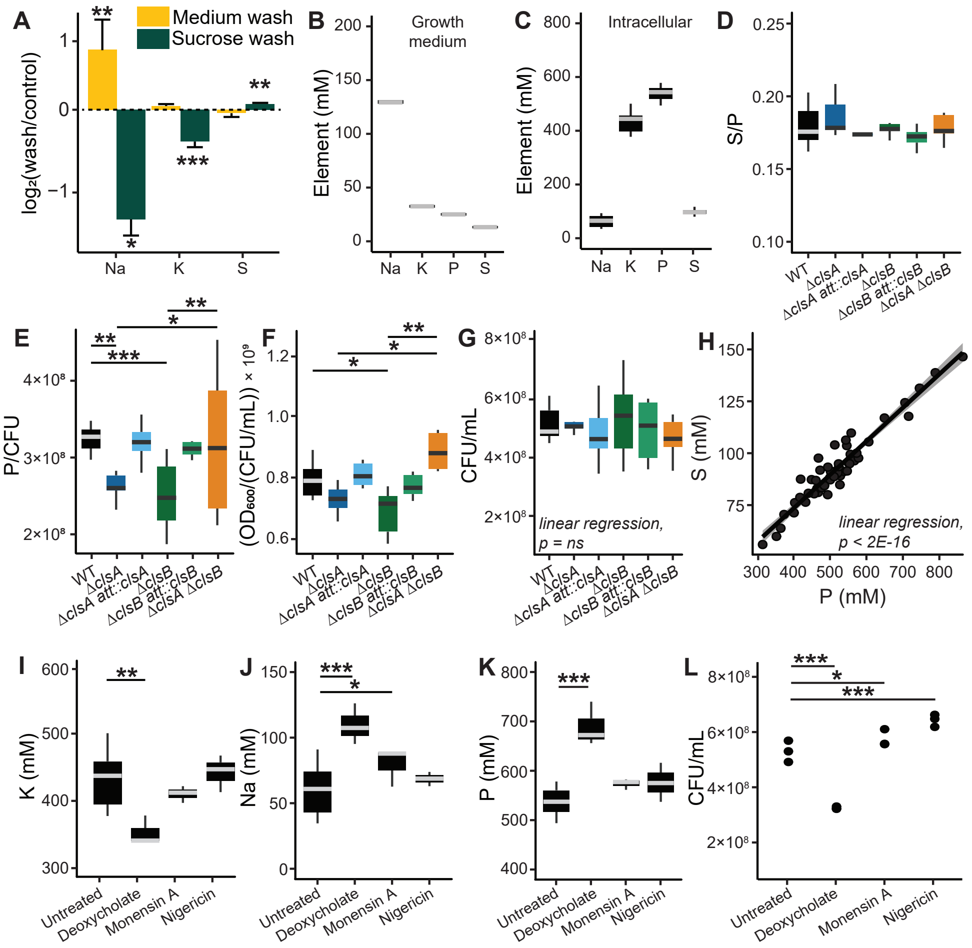

Figure S5. Washing but not loss of *cls* genes disrupts intracellular elemental concentrations. Inductively coupled plasma mass spectrometry (ICP-MS) analysis using wash-free sample preparation to quantify intracellular elemental concentrations. **A.** $\log_2$(fold change) of elements comparing unwashed cells to cell pellets washed with either BHIS growth medium or an isotonic sucrose solution. Error bars indicate standard deviation. **B.** Concentrations of detected elements in BHIS growth medium. **C.** Intracellular concentrations of detected elements in WT *B. fragilis* P207. **D.** Intracellular sulfur in *B. fragilis* P207 strains normalized to P. **E.** Intracellular phosphorus (P) per 10^6 colony-forming units (CFU) in *B. fragilis* P207 wild-type (WT), Δ*clsA*, Δ*clsB* and Δ*clsA* Δ*clsB* strains. **F.** OD_{600} normalized to CFU per mL of strain cultures used in ICP-MS input. **G.** Raw CFU/mL of each strain shown in panels F and G. **H.** Intracellular P content compared to sulfur (S) content across *cls* strains and treatment conditions. **I., J.** Intracellular concentrations of (**I.**) K^+ and (**J.**) Na^+ after a 20 min exposure to 0.01% deoxycholate, 0.8 µg/mL monensin A and 0.1 µg/mL nigericin. Each treatment had the same culture input. **K.** Intracellular P concentrations of *B. fragilis* WT cells

bioRxiv preprint doi: https://doi.org/10.1101/2025.05.12.653583; this version posted May 19, 2025. The copyright holder for this preprint (which was not certified by peer review) is the author/funder, who has granted bioRxiv a license to display the preprint in perpetuity. It is made available under aCC-BY 4.0 International license.

after 20 min of exposure to the indicated stress conditions. **L.** CFU/mL of strain cultures after exposure to the treatments in **I–L**. For all boxplots, the gray or black middle lines indicate the median; the top and bottom edges of the box indicate the 25th and 75th percentiles of n = 3 replicates for wash and treatment experiments and n = 10 replicates for rest. Whiskers indicate 1.5× the interquartile range (IQR). For the linear regression, the shade ribbon indicates standard error of the mean. All statistical comparisons were made using linear regression, with unadjusted p-values shown; *, $p < 0.05$; **, $p < 0.01$; ***, $p < 0.001$.

bioRxiv preprint doi: https://doi.org/10.1101/2025.05.12.653583; this version posted May 19, 2025. The copyright holder for this preprint (which was not certified by peer review) is the author/funder, who has granted bioRxiv a license to display the preprint in perpetuity. It is made available under aCC-BY 4.0 International license.

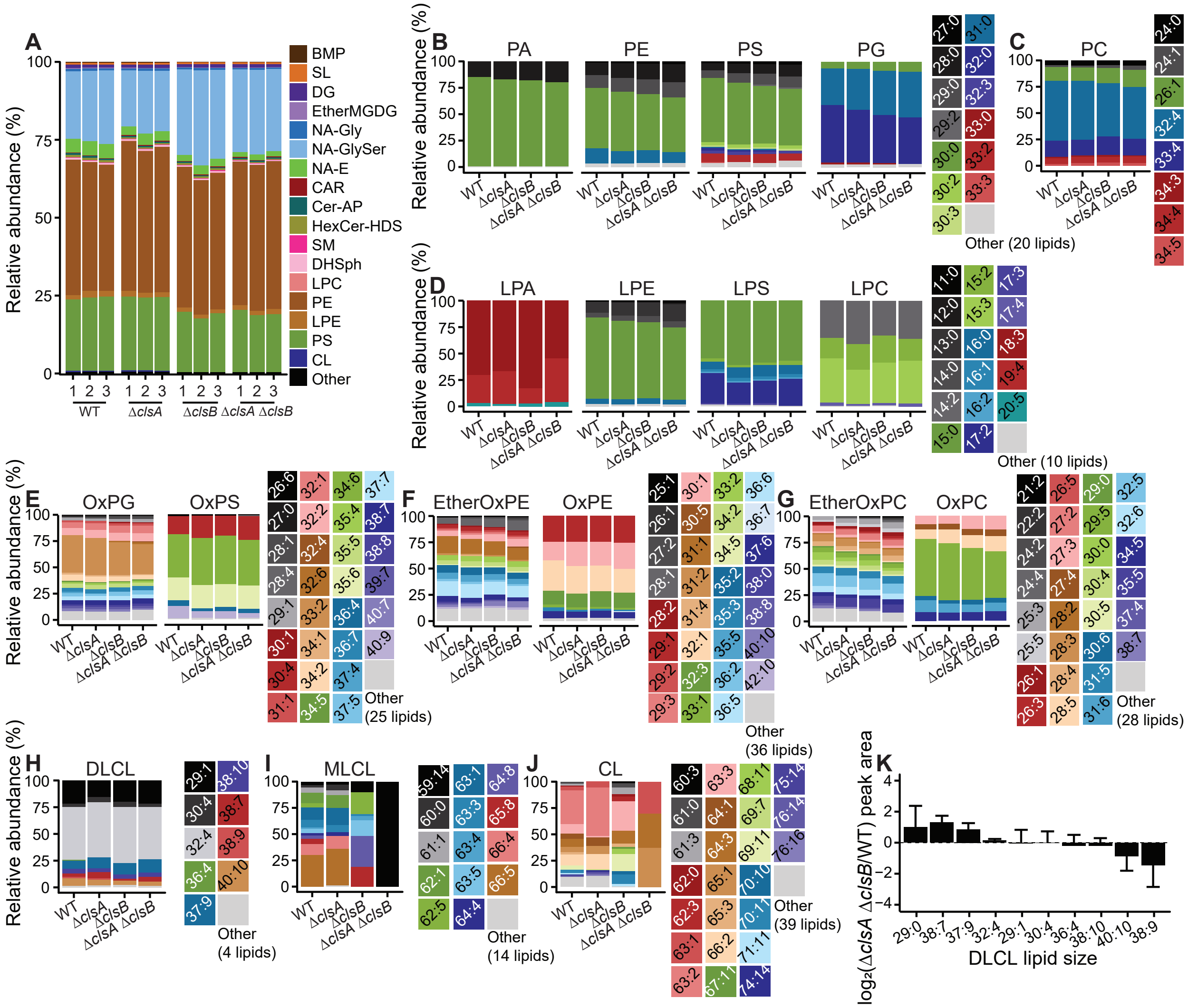

Figure S6. Cardiolipin synthases shape the membrane lipid population. A-J. Relative abundance of lipid species in membranes across *B. fragilis* WT, Δ*clsA*, Δ*clsB*, and Δ*clsA* Δ*clsB* strains in positive (**A.**) or negative (**B-J.**) electrospray ionization modes for UHPLC–MS/HRMS. For **A,** colors designate lipid family. Positive-mode lipid families are abbreviated as follows: BMP, bismonoacylglycerophosphate; SL, saccharolipids; DG, diacylglycerol; EtherMG, ether-linked with monogalactosyl/monoglucosyl conjugation; NA, *N*-acyl, with substituent conjugations indicated for glycyl- (Gly), glycylserine- (GlySer), and ethanolamine- (E); CAR, acylcarnitine; CER, ceramide, AP indicating alpha-hydroxy fatty acid-phytospingosine and Hex indicating hexosyl-; SM, sphingomyelin; DHSph, sphinganine (please see the legend for panel E for other abbreviations). For **B-J**, species are colored based on the total number of

bioRxiv preprint doi: https://doi.org/10.1101/2025.05.12.653583; this version posted May 19, 2025. The copyright holder for this preprint (which was not certified by peer review) is the author/funder, who has granted bioRxiv a license to display the preprint in perpetuity. It is made available under aCC-BY 4.0 International license.

carbons in their acyl chains. Lipids at <1% abundance are colored gray, with the number of lipid species in this category indicated in the figure. Relative abundance plots are grouped as (**B.**) two or (**C.**) one acyl-tailed phospholipid species, with oxidized and ether-linked lipids shown in **E.-G.**, or (**H.**) dilysocardiolipin, (**I.**) monolysocardiolipin or (**J.**) cardiolipin species. Negative-mode lipid families are abbreviated as follows: PA, phosphatidic acid; PE, phosphatidylethanolamine; PS, phosphatidylserine; PG, phosphatidylglycerol; PC, phosphatidylcholine; CL, cardiolipin. L-, ML- and DL- indicate lyso-, monolyso- and dilyso-, respectively. Ox and Ether indicate oxidized and ether-linked, respectively. **K.** $\log_2$(fold change) peak area of dilysocardiolipin species comparing Δ*clsA* Δ*clsB* to WT (linear regression, $p < 0.00029$; $\log_{10}$[peak area] ~ strain + DLCL species). Error bars indicate standard deviation.

bioRxiv preprint doi: https://doi.org/10.1101/2025.05.12.653583; this version posted May 19, 2025. The copyright holder for this preprint (which was not certified by peer review) is the author/funder, who has granted bioRxiv a license to display the preprint in perpetuity. It is made available under aCC-BY 4.0 International license.

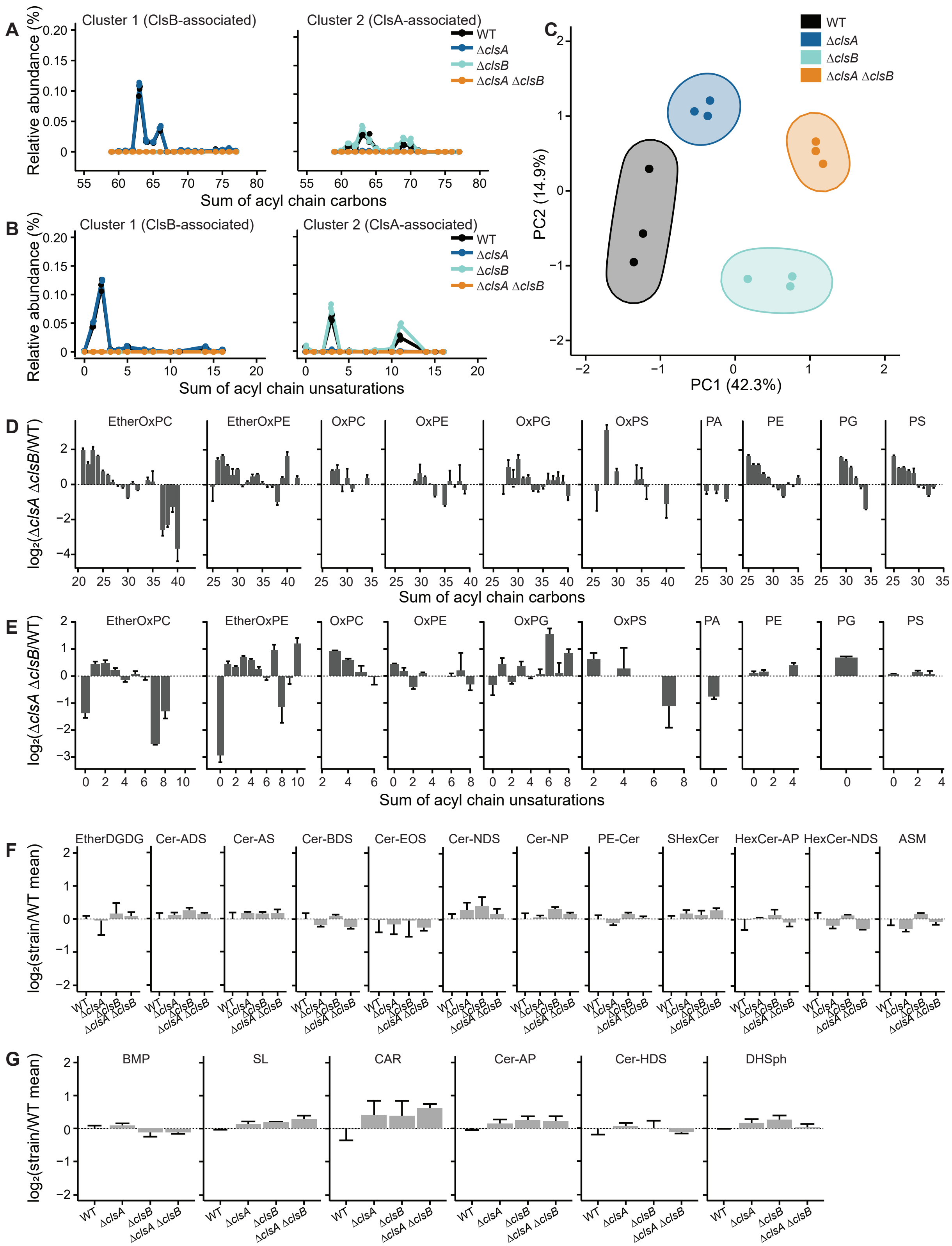

1356

1357

bioRxiv preprint doi: https://doi.org/10.1101/2025.05.12.653583; this version posted May 19, 2025. The copyright holder for this preprint (which was not certified by peer review) is the author/funder, who has granted bioRxiv a license to display the preprint in perpetuity. It is made available under aCC-BY 4.0 International license.

Figure S7. Cardiolipin synthases produce unique lipid products and their loss remodels overall membrane lipid composition. A., B. Relative abundance of lipids from K-means clusters 1 and 2 across *B. fragilis* WT, Δ*clsA*, Δ*clsB* and Δ*clsA* Δ*clsB* strains plotted by (**A.**) acyl chain length or (**B.**) acyl chain unsaturations. **C.** Principal component analysis plot of lipids in the *B. fragilis* membrane excluding cardiolipin and monolysocardiolipin species. Minimum volume-enclosing ellipses were estimated using the Khachiyan algorithm. **D., E.** $\log_2$(fold change) of Pa, PE, PG and PS lipids in Δ*clsA* Δ*clsB* cells compared to WT plotted by the sum of acyl chain (**D.**) carbons or (**E.**) unsaturations. **F., G.** $\log_2$(fold change) of lipids detected in (**F.**) negative or (**G.**) positive ionization modes with values for each strain compared to the mean abundance in WT cells. See figures 7 and S6 for lipid family abbreviations. Error bars indicate standard deviation. Unadjusted p-values are shown.

bioRxiv preprint doi: https://doi.org/10.1101/2025.05.12.653583; this version posted May 19, 2025. The copyright holder for this preprint (which was not certified by peer review) is the author/funder, who has granted bioRxiv a license to display the preprint in perpetuity. It is made available under aCC-BY 4.0 International license.

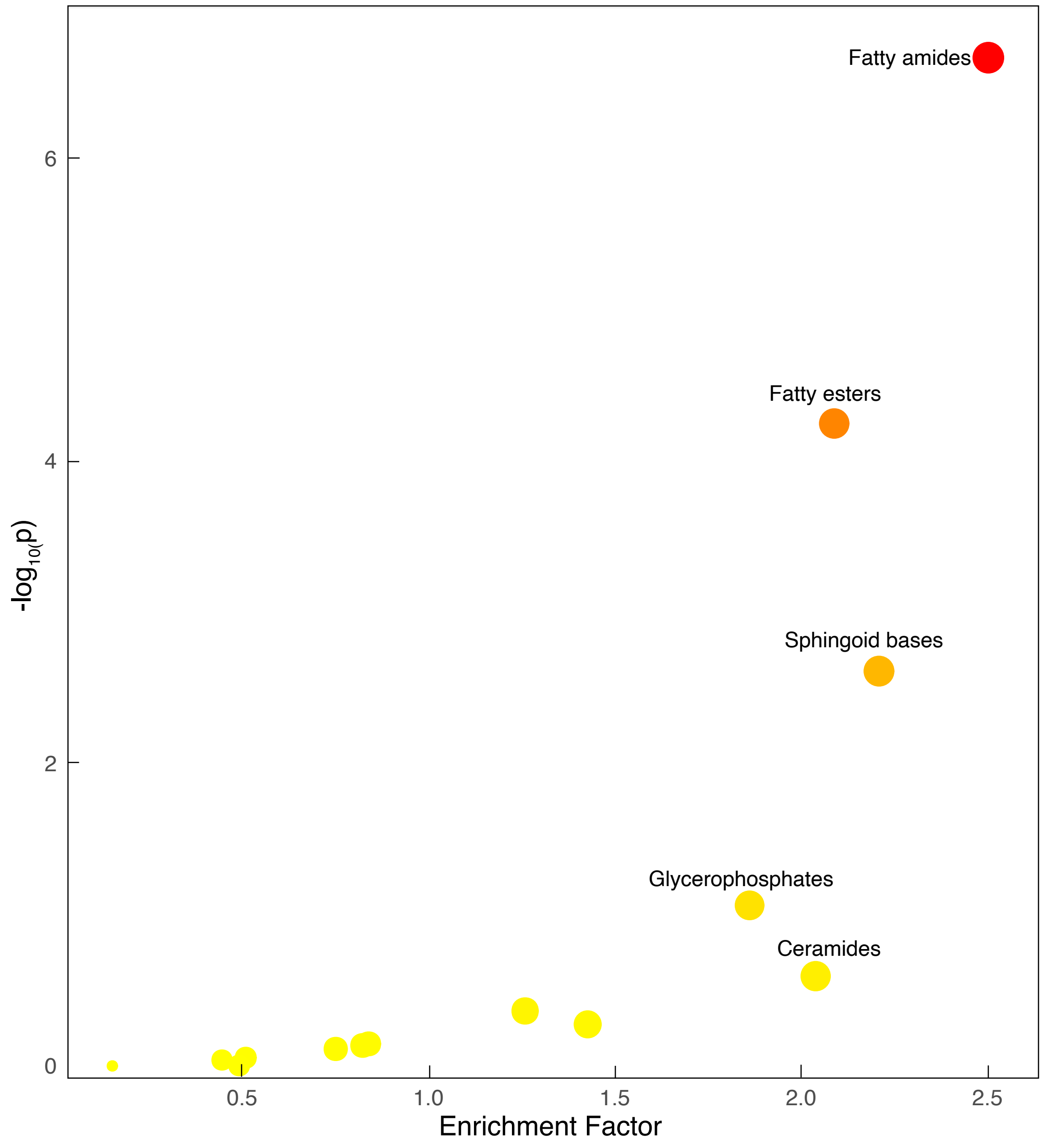

Figure S8. Pathway enrichment analysis for main lipid categories. Pathway analysis is performed with MetaboAnalyst 6.0, with default mummichog parameters with p-value cutoff of 0.0005 and lipid – main chemical class as the selected metabolite sets containing at least 3 entries.

bioRxiv preprint doi: https://doi.org/10.1101/2025.05.12.653583; this version posted May 19, 2025. The copyright holder for this preprint (which was not certified by peer review) is the author/funder, who has granted bioRxiv a license to display the preprint in perpetuity. It is made available under aCC-BY 4.0 International license.

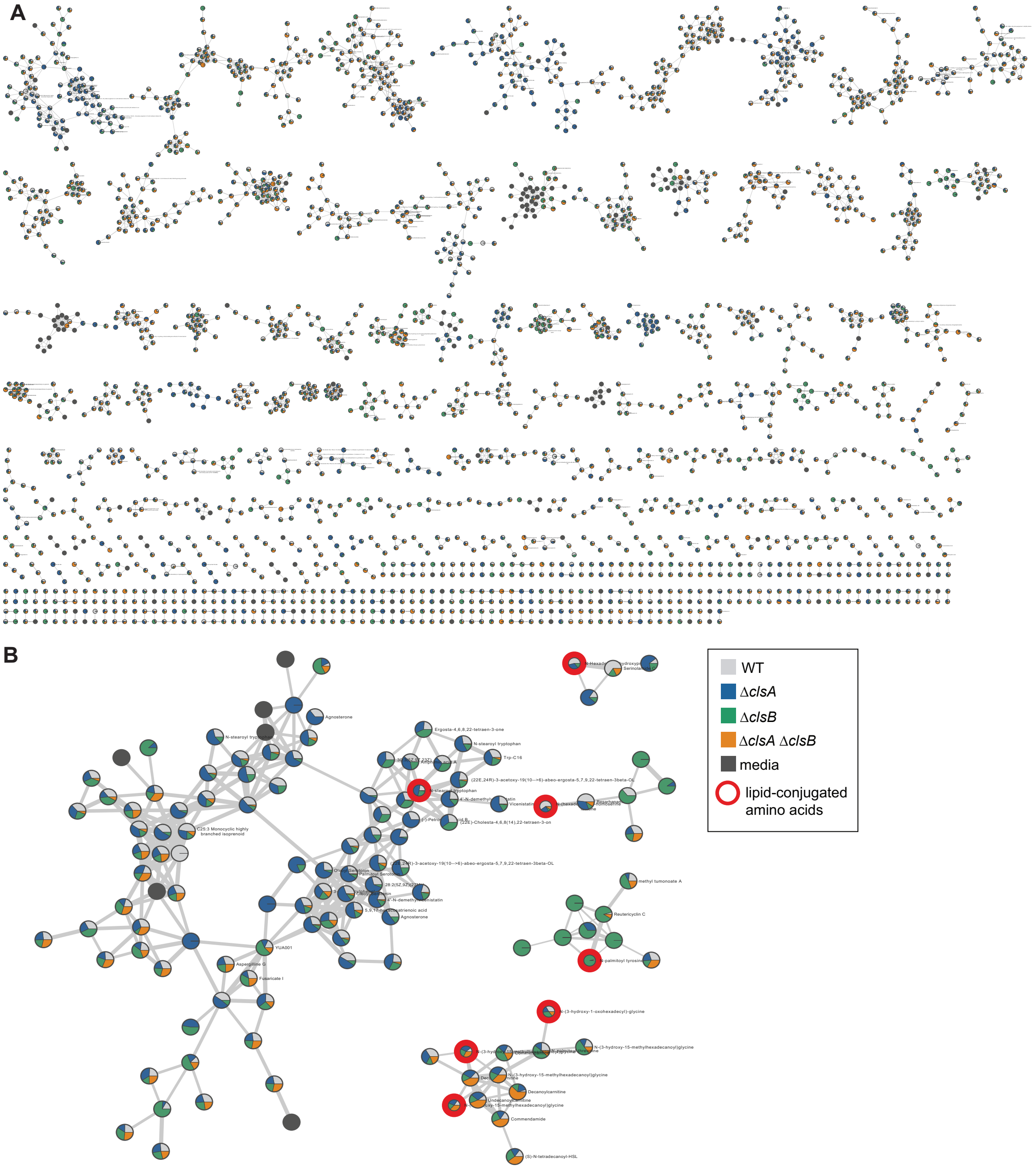

Figure S9. Molecular networking shows chemically related metabolites in *B. fragilis* strains. A. Feature-based molecular networking of all features (excluding singletons) from WT, Δ*clsA*, Δ*clsB*, Δ*clsA* Δ*clsB*, and extracted BHIS media. Each feature is a node, connected by an edge calculated from cosine

bioRxiv preprint doi: https://doi.org/10.1101/2025.05.12.653583; this version posted May 19, 2025. The copyright holder for this preprint (which was not certified by peer review) is the author/funder, who has granted bioRxiv a license to display the preprint in perpetuity. It is made available under aCC-BY 4.0 International license.

similarity score. **B.** Subset of features connected by an edge to fatty amides highlighted in Fig. 8 (red border). Each pie chart in the node is colored by relative abundance of the feature in each strain.

Table S1. Primers, vectors and strains used in this study.

Table S2. Metadata for genomics analyses.

Table S3. Raw data for the lipidomics analyses.

Table S4. Raw data for the ICP-MS analyses.

Table S5. Raw data for untargeted metabolomics analyses.

Table S6. Pathway enrichment analysis for main lipid categories.

Table S7. Pathway enrichment analysis for sub non-lipid categories.
